# TMT-Based Multiplexed (Chemo)Proteomics on the Orbitrap Astral Mass Spectrometer

**DOI:** 10.1016/j.mcpro.2025.100968

**Published:** 2025-04-08

**Authors:** Yuchen He, Ka Yang, Shaoxian Li, Martin Zeller, Graeme C. McAlister, Hamish I. Stewart, Christian Hock, Eugen Damoc, Vlad Zabrouskov, Steven P. Gygi, Joao A. Paulo, Qing Yu

**Affiliations:** 1 Department of Cell Biology, Harvard Medical School, Boston, Massachusetts, United States; 2 Department of Biochemistry and Molecular Biotechnology, University of Massachusetts Chan Medical School, Worcester, Massachusetts, United States; 3 Thermo Fisher Scientific, Bremen, Germany; 4 Thermo Fisher Scientific, San Jose, California, United States

**Keywords:** TMT, DDA, DIA, Orbitrap Astral mass spectrometer, ABPP

## Abstract

Ongoing advancements in instrumentation has established mass spectrometry (MS) as an essential tool in proteomics research and drug discovery. The newly released Asymmetric Track Lossless (Astral) analyzer represents a major step forward in MS instrumentation. Here, we evaluate the Orbitrap Astral mass spectrometer in the context of tandem mass tag (TMT)-based multiplexed proteomics and activity-based proteome profiling, highlighting its sensitivity boost relative to the Orbitrap Tribrid platform—50% at the peptide and 20% at the protein level. We compare TMT data-dependent acquisition and label-free data-independent acquisition on the same instrument, both of which quantify over 10,000 human proteins per sample within 1 h. TMT offers higher quantitative precision and data completeness, while data-independent acquisition is free of ratio compression and is thereby more accurate. Our results suggest that ratio compression is prevalent with the high-resolution MS2-based quantification on the Astral, while real-time search-based MS3 quantification on the Orbitrap Tribrid platform effectively restores accuracy. Additionally, we benchmark TMT-based activity-based proteome profiling by interrogating cysteine ligandability. The Astral measures over 30,000 cysteines in a single-shot experiment, a 54% increase relative to the Orbitrap Eclipse. We further leverage this remarkable sensitivity to profile the target engagement landscape of FDA-approved covalent drugs, including sotorasib and adagrasib. We herein provide a reference for the optimal use of the advanced MS platform.

## Highlights

• Astral TMT quantifies 50% more peptides and 20% more proteins than Tribrid.
• Both TMT and DIA quantify >10,000 proteins in 1 hr per sample on the Astral.
• Astral TMT MS2 gives better precision, while DIA offers greater quant accuracy.
• TMT RTS-MS3 offers best precision, higher accuracy, and success-detecting changes.
• TMT-ABPP on Astral profiles 30,000+ reactive cysteines, revealing more drug targets.

## Graphical Abstract

**Figure:**
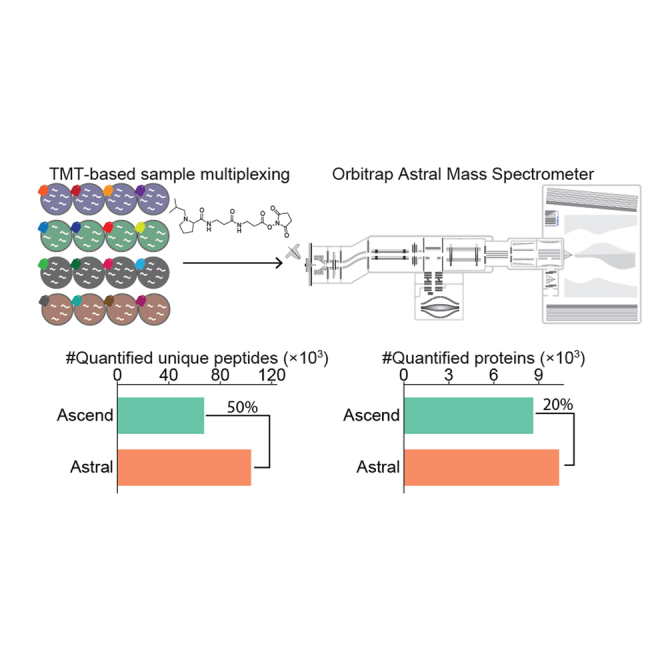

## In Brief

The Orbitrap Astral boosts TMT-based quantitative proteomics, quantifying 50% more peptides and 20% more proteins than the Orbitrap Tribrid platform. TMT quantifies over 10,000 proteins per sample in 1 h—comparable to label-free DIA—while offering superior precision and completeness. Although ratio compression persists, RTS-MS³ on the Tribrid alleviates it. TMT-ABPP on the Astral enables single-shot analysis of over 30,000 cysteines—a 54% increase over the Tribrid—revealing new drug targets. This work optimizes TMT-based proteomics workflows on the Astral.

Mass spectrometry-based proteomics is rapidly evolving, and a variety of data collection strategies have been developed, among which data-dependent acquisition (DDA) and data-independent acquisition (DIA) represent two key strategies for generating reliable and reproducible data (1, 2). The evolution of these methodologies has significantly transformed proteomics. DDA has traditionally been favored for its ability to provide high-confidence peptide identification and compatibility with sample multiplexing techniques, such as tandem mass tag (TMT)-based isobaric labeling, while DIA has gained traction due to its improved reproducibility and low cost.

Sample multiplexing-based techniques have been developed and widely adopted to improve throughput and reduce variability as many samples can be analyzed simultaneously (3, 4, 5, 6). These strategies enable deep analysis with essentially no missing values within the multiplexed set of samples. Among many labeling reagents, TMT routinely allows for simultaneous quantification of proteins across up to 35 samples, providing high quantitative precision and throughput (6). One caveat of TMT-based proteomics is that the quantification accuracy is negatively impacted by co-isolated interfering ions, resulting in ratio compression. However, the ratio compression can be effectively reduced by using the SPS-MS3 strategy (7).

Recent advancements in mass spectrometry technology, particularly the development of the Orbitrap Astral mass spectrometer, have introduced paradigm-shifting capabilities in proteomic analyses (8, 9, 10, 11, 12). The Orbitrap Astral, featuring a quadrupole mass filter, an Orbitrap analyzer, and an Asymmetric Track Lossless (Astral) analyzer, facilitates the rapid acquisition of high-resolution accurate mass MS2 spectra, addressing the growing demand for high-throughput and ultra-sensitive proteomics. Notable examples include clinical plasma proteomics (13) and single-cell proteomics (14). The rapid scan speed of the Orbitrap Astral significantly elevates label-free DIA proteomic analysis, allowing for extensive proteome coverage and high-quality data generation within shorter analysis times (8, 9, 10, 11, 12). However, the potential of the Orbitrap Astral in isobaric labeling-based multiplexed proteomics has not been thoroughly explored.

Here, we evaluate TMT-based quantitative proteomics on the Orbitrap Astral and compare TMT HRMS2-DDA with label-free DIA on the same instrument. By profiling the proteomes from four different cell lines, TMT-based DDA and label-free DIA experiments achieve similar coverage of over 10,000 human proteins within 1 h per proteome. HRMS2-TMT exhibits higher quantitative precision and data completeness. However, it is subject to ratio compression and consequently shows lower accuracy. The ratio compression arising from co-isolated ion species is demonstrated further using a two-proteome model where substantial interference is introduced. We further leverage the significantly improved sensitivity on the Astral to conduct TMT-based activity-based proteome profiling (ABPP) by globally interrogating reactive cysteines. Over 30,000 reactive cysteines are measured in a single-shot experiment, representing a 54% increase relative to the Orbitrap Eclipse (15). We showcase the TMT-ABPP platform by characterizing the target engagement landscape of two FDA-approved covalent drugs, sotorasib and adagrasib. The study highlights the advantages of TMT-based proteomics and ABPP on the Orbitrap Astral in achieving extensive proteome coverage and producing high-quality data as well as limitations including ratio compression.

## Experimental Procedures

### Experimental Design and Statistical Rationale

This study presents an evaluation of TMT-based multiplexed proteomics and chemoproteomics on the Orbitrap Astral mass spectrometer. Experimental designs are illustrated in figure panels and described at the start of each subsection in the results. Briefly, for the four-cell-line experiments, the goal was to benchmark the Orbitrap Astral’s identification and quantification performance using a model system. The four-cell-line system was made into two versions. First, the advanced model system contained three biological replicates for the cell lines (HEK293T, U2OS, HCT116, and RPE1), and each biological replicate of HEK293T and HCT116 was split into two technical replicates. The goal is to evaluate how reproducible the measurement is between two technical replicates and establish filters for precise quantification. Second, the standard model system contained four biological replicates for the cell lines (RPE1, U2OS, HEK293T, U2OS, and HCT116) and used to benchmark performance on different MS platforms. For the two-proteome interference model, human and yeast peptides were mixed at a 20:1 ratio (w/w). The low-abundance yeast peptides had variable ratios (1, 1.5, and 2, each with five replicates). This was to mimic a highly complex biological sample where only a small fraction of proteins change. Both TMT-labeled and label-free samples were prepared for the four-cell-line model system and the two-proteome interference model in order to benchmark TMT- and DIA-based quantification. To benchmark TMT-based reactive cysteine profiling on the Astral, HEK293T cell lysates with varying concentrations of scout fragments for dose response. The same method was applied to profile off-targets of KRAS C12 inhibitors using HCC44 and MIAPACA2 cell lysates.

### Cell Culture

Human RPE1 (#CRL-4000), U2OS (#HTB-96), HCT116 (#CCL-247), and HEK293T (#CRL-3216) cells were purchased from the American Type Culture Collection and grown in Dulbecco's modified Eagle's medium supplemented with 10% fetal bovine serum and 1% penicillin/streptomycin until 80% confluent. Baker's yeast, *Saccharomyces cerevisiae*, was grown in the yeast extract-peptone-dextrose broth until *A*_*600*_ = 0.8. Cells were washed twice with ice cold PBS, pelleted, and stored at −80 °C until use.

### Whole Proteome Sample Preparation

Human cells were lysed by resuspension in lysis buffer consisting of 8M urea and 200 mM 4-(2-hydroxyethyl)-1-piperazinepropanesulfonic acid (EPPS) pH 8.5 with protease and phosphatase inhibitors (Pierce A32953 and A32957), followed by 10 passes through a 21-gauge syringe. Yeast cells were lysed by bead-beating in the same lysis buffer above. Protein concentration was determined with the BCA assay. Lysates were reduced with 5 mM tris(2-carboxyethyl)phosphine for 15 min at room temperature and alkylated with 10 mM iodoacetamide for 30 min at room temperature in the dark. Excess iodoacetamide was quenched with 10 mM dithiothreitol (DTT) for 15 min at room temperature. Proteins were isolated by chloroform-methanol precipitation, subsequently resuspended in 200 mM EPPS pH 8.5 (∼1 mg/ml) and digested first with LysC for 12 h at room temperature shaking on a vortexer followed by a 6 h digestion at 37 °C with trypsin. Protein digests were split in half, with one half labeled with TMT reagents while the other left unlabeled for label-free analysis. The TMT-labeled peptides were then mixed and desalted by solid phase extraction (SepPak cartridge, Waters) prior to basic pH fractionation or LC-MS analysis.

### In-Lysate TMT-Based Reactive Cysteine Profiling

The streamlined reactive cysteine profiling was performed as described previously (15, 16). Twenty microgram per soluble HEK293T native lysate in 10 μl of lysis buffer (PBS, pH 7.4, 0.1% NP-40) was loaded into a 96-well plate. The samples were treated by compounds (the final concentrations were described in the figure legend) and then 500 μM desthiobiotin iodoacetamide each for 1 h at room temperature. Next, 3 μl of SP3 beads (a 1:1 mixture of hydrophobic and hydrophilic types, 50 mg/ml, Cat. #45152105050250 and Cat. #65152105050250) and 30 μl of ∼98% ethanol supplemented with 20 mM DTT were added to the plate. The plate was incubated for 15 min with mild shaking, then placed on a magnetic stand, and the liquid was aspirated. The beads were washed once with 150 μl of 80% ethanol and resuspended in 30 μl of Hepes buffer (Ph 7.4, 0.1% Triton-X100) supplemented with 20 mM iodoacetamide. This mixture was incubated in the dark for 30 min with vigorous shaking. Subsequently, 60 μl of ∼98% ethanol supplemented with 20 mM DTT was added, followed by bead-based clean-up with two additional washes using 80% ethanol.

Next, 20 μl of 200 mM EPPS buffer (pH 8.5) containing 0.2 μg Lys-C was added to the beads. After a 3-h incubation at room temperature, 10 μl of 200 mM EPPS buffer containing 0.2 μg trypsin was added and incubated with the beads at 37 °C overnight. Then 10 μl of acetonitrile and 4 μl of TMT (12.5 μg/μl) reagent were added, followed by gentle mixing at room temperature for 60 min. The reaction was quenched by adding 7 μl of 5% hydroxylamine, and all TMT-labeled samples were combined into a multiplexed sample. After being dried using a SpeedVac and then desalted using a 100-mg Sep-Pak column, the desalted peptides were resuspended in 310 μl of 100 mM Hepes buffer (pH 7.4), and 50 μl of Pierce High Capacity Streptavidin Agarose (Cat. #20359) was added. This mixture was incubated at room temperature for 3 h. The resulting mixture was then loaded onto an AcroPrep plate with a PTFE filter (0.22 μm pore size) and centrifuged at 100*g* for 30 s. The beads were washed sequentially with 200 μl of 100 mM Hepes (pH 7.4) with 0.05% NP-40 twice, 200 μl of 100 mM Hepes (pH 7.4) three times, and 200 μl of water twice. The peptides were eluted sequentially using the following: (1) elution buffer (80% acetonitrile, 0.1% formic acid) with 15-min incubation at room temperature twice and (2) elution buffer heated to 72 °C with 10-min incubation twice. The combined eluate was dried in a SpeedVac and desalted *via* StageTip prior to LC-MS analysis.

### LC-MS

LC separation was conducted using either an EASY-nLC 1200 System (Thermo Scientific) or a Vanquish Neo System (Thermo Scientific) with a custom-packed Accucore 150 resin column (30 cm length × 100 μm inner diameter). The mobile phase A consisted of 0.1% formic acid in 5% acetonitrile. Mobile phase B consisted of 0.1% formic acid in 95% acetonitrile. Eluted peptides were ionized *via* electrospray ionization and subsequently analyzed using an Orbitrap Astral mass spectrometer (Thermo Scientific), an Orbitrap Eclipse Tribrid mass spectrometer (Thermo Scientific), or an Orbitrap Ascend Tribrid mass spectrometer (Thermo Scientific). The spray voltage was maintained at 2.2 kV, with the ion transfer tube temperature set to 290 °C, and the source RF set to 50.

In label-free single-shot proteomics with DIA, the Orbitrap MS1 scan was performed at a resolution of 240,000 (at m/z 200) with a scan range of 380 to 980 m/z and an AGC target of 500%. Precursor ions were isolated using a quadrupole mass filter with a 2 m/z isolation width and fragmented by higher-energy collisional dissociation (HCD) at a normalized collision energy (NCE) of 25% or 27%. For Astral MS2 scans, the AGC target was 500% with a maximum injection time of 3 ms.

In TMT-based proteomics with DDA, the Orbitrap MS1 scan was performed at a resolution of 60,000 (at m/z 200), with a scan range of 400 to 1500 m/z and an AGC target of 200%. Precursor ions were isolated using a quadrupole with a 0.5 m/z isolation width and fragmented by HCD at an NCE of 36%. For Astral MS2 scans, the AGC target was 100%, with a maximum injection time of 10 ms if precursors were dynamically excluded after three MS2 samplings or 20 ms if excluded after one MS2 sampling. For Orbitrap MS2 scans, the AGC target was 500%, with a resolution of 45,000 and a maximum injection time of 86 ms. For the real-time search-based MS3 (RTS-MS3) method on the Orbitrap Eclipse, precursor ions were isolated using a quadrupole with a 0.5 m/z isolation width and fragmented by collision-induced dissociation at an NCE of 34%, an activation time of 10 ms, and an activation Q of 0.25. The AGC target for the ion trap was 100%, with a maximum injection time of 50 ms. For RTS settings, trypsin/P digestion was used for a concatenated forward-reverse database. Cysteine carbamidomethylation and TMTpro modifications on lysine and peptide N-termini were set as static modification, while methionine oxidation was set as a variable modification. The search was limited to 50 ms with Xcorr of 1.4, dCn of 0.1, precursor ppm of 20, and charge state of 2. Matches to database entries with “##” (reverse) and “contaminant” were excluded. MS3 excluded peaks 50 m/z below the precursor m/z and 5 m/z above, also excluding TMTpro tag loss ions. Up to 10 SPS ions were selected, fragmented by HCD at an NCE of 55%, and MS3 spectra were collected in the Orbitrap with a resolution of 50,000; AGC target of 500%; and maximum injection time of 200 ms.

### Data Process

DIA data analysis was performed using FragPipe (v21.0) or standalone DIA-NN (v1.8.1) (17, 18, 19, 20). TMT data analysis was performed using a Comet-based in-house pipeline. All settings were kept default. Two human protein databases (proteome ID: UP000005640) were used, including reviewed (Swiss-Prot), reviewed (Swiss-Prot) isoforms, and unreviewed (TrEMBL) proteins. The database used for the four-cell data was downloaded on November 24, 2021, and contained 101,029 entries. The database for the remaining data was downloaded on March 15, 2024, and contained 104,557 entries. For DIA data, the number of missed cleavages permitted is 1. Fixed modification was set to carbamidomethylation on cysteine (+57.02146). Variable modifications were set as oxidation on methionine (+15.9949, max allowed occurrence 1) and acetylation on the N-term (+42.0106, max allowed occurrence 1). Mass tolerances for both precursor and fragment ions were set to 20 ppm for initial peak matching in FragPipe. Both FragPipe and DIA-NN performed automatic inference of the mass tolerance then. False discovery rates were set to 1% at both peptide and protein levels. TMT data were filtered by excluding peptide-spectrum matches (PSMs) with mean signal-to-noise (S/N) ratio below 80 (sum S/N below 1440 for 18plex) or any reporter resolution below 45,000 when the corresponding S/N was above 200. The mean S/N of 80 was established for the Astral to achieve variability levels comparable to those observed with the Eclipse, as illustrated in supplemental Fig. S3. The resolution of 45,000 was determined to achieve baseline separation of the n and c pairs of the reporter ions of TMTpro 18plex (supplemental Note S1). Data formatting and visualization was performed using either base R (21) or functions in the package ggplot2 (22). The R package plot3D (23) was used to generate the 3D density plot. Student *t* test was performed to generate *p* values in cysteine profiling experiments.

## Results and Discussion

### Signal and Resolution Filters for TMT-Based Quantification

**Fig. 1:**
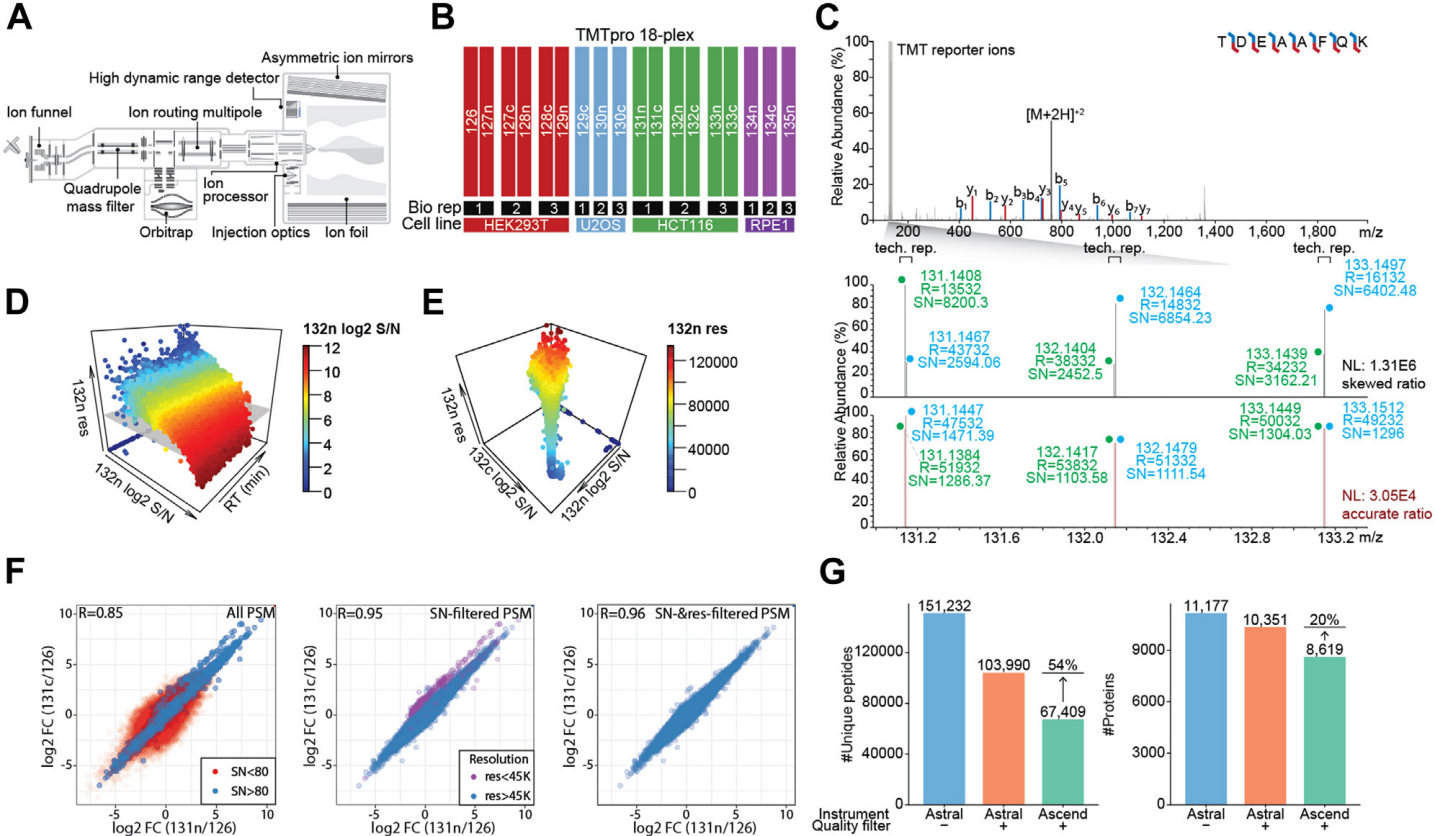
**Applying signal and resolution filters for improved TMT-based proteomic data quality.***A*, the Orbitrap Astral instrument schematic. *B*, experimental setup. Each cell line was cultured in three biological replicates. Each biological replicate of HEK293T and HCT116 was split into two technical replicates. *C*, MS2 spectrum of the representative peptide collected using the Astral analyzer. This annotated tandem spectrum was created using the Interactive Peptide Spectrum Annotator (IPSA). *D*, 3D plot showing resolution *versus* S/N and retention time for TMT 132n channel. *E*, 3D plot showing resolution *versus* S/N of TMT 132n channel and S/N of the technical replicate TMT 132c channel. *F*, scatter plot showing correlation between two technical replicates, without filter (*left panel*), with S/N filter alone (*middle panel*), and with both S/N and resolution filters (*right panel*) applied. *G*, bar plot showing numbers of unique peptides and proteins from the data collected by the Astral and the Ascend with or without the quantification filter. Astral, asymmetric track lossless; S/N, signal-to-noise; TMT, tandem mass tag.

TMT-based proteomics is a powerful technique for identifying and quantifying proteins concurrently across multiple complex biological samples. Using this method, TMT-labeled peptides from different samples are pooled and analyzed together. Sample-specific TMT reporter ions are released upon fragmentation, enabling relative quantification of peptides. We postulate that the performance of TMT-based proteomics can benefit from the Orbitrap Astral mass spectrometer, as the Astral analyzer offers high scan speed, resolution, and sensitivity (Fig. 1*A*) (8). As a multireflection mass analyzer, resolution in Astral is subject to space charge effects (24). An inherent challenge lies in accumulating more signal for better quantification while maintaining resolution for TMT reporter ions which differ by only a few milli-Dalton (mDa) (4, 5, 6). We created an 18-plex sample which included both technical and biological replicates to evaluate the space charge effect and establish a quantitative filter (Fig. 1*B* and supplemental Data S1). The sample was fractionated into 12 fractions, and each fraction was analyzed with a 90-min HRMS2 method. Figure 1*C* presents an interactive peptide spectrum annotator-generated (25) example of a single scan MS2 spectrum of the TMT-labeled peptide TDEAAFQK. TMT131n-131c, 132n-132c, and 133n-133c are technical replicate pairs from HCT116. In one scan of the peptide (Fig. 1*C*, lower panel), the expected 1:1 ratio was measured. The average S/N of these reporter ions was around 1200. In another scan from the same peptide, we observed distorted ratios between these TMT reporter pairs due to space charging, which is evident by the reduced resolving power with an average S/N of 5000 (Fig. 1*C* upper panel). The space charging and the resulting low resolution were observed across the entire LC-MS analysis (Fig. 1*D*). This suggests that scans with an excessive signal of TMT ions must be removed from the final result. In contrast, PSMs with inadequate number of TMT ions showed more variability, even though higher resolutions were obtained (Fig. 1*E*).

This trade-off was further investigated by (1) varying the Astral MS2 AGC target at 25%, 50%, 100%, and 200%, when loading 500 ng yeast triple knockout (26) peptides on the column (supplemental Figs. S1 and S2) varying the triple knockout peptide loading amount at 125 ng, 250 ng, 500 ng, and 1000 ng on the column, while keeping the Astral MS2 AGC target at 100% (supplemental Fig. S2). When varying MS2 AGC target, we observed that the overall distribution of the 127n reporter ion resolution shifted lower as the AGC target increases (supplemental Fig. S1*A*). Although higher AGC targets yielded improved S/N, a greater number of PSMs exhibited 127n resolutions below 45,000 (supplemental Fig. S1*B*). Conversely, reducing the AGC target enhanced the reporter ion resolution, but the coefficient of variation (CV) increased due to the reduced signal (supplemental Fig. S1*C*). When varying peptide loading amount, we observed that lower peptide loads reduced the percentage of PSMs exhibiting low reporter ion resolutions (below 45,000) (supplemental Fig. S2*A*). This is attributed to reduced space charging and improved reporter ion resolution. Conversely, lower peptide load also increased the proportion of PSMs with insufficient reporter ion S/N (below 80 per TMT reporter channel) (supplemental Fig. S2*B*). Overall, we observed a slight increase in the percentage of PSMs with either low reporter ion resolution or low reporter ion S/N as the loading amount decreased (supplemental Fig. S2*C*). In addition, the total number of PSMs decreased when lowering loading amount.

To achieve accurate quantification, we implemented quality filters by excluding PSMs with mean S/N ratio below 80 (sum S/N below 1440 for 18plex) or any reporter resolution below 45,000 when the corresponding S/N was above 200 (Fig. 1*F*). The mean S/N of 80 was established for the Astral to achieve variability levels comparable to those observed with the Eclipse, as illustrated in supplemental Fig. S3. The resolution of 45,000 was determined to achieve baseline separation of the n and c doublets of TMTpro reporter ions (supplemental Note S1). These quality filters were applied to all the following experiments. Prior to applying these filters, the Pearson correlation coefficient (R) between technical replicates (TMT131n and 131c) was 0.85; after applying the filters, this value increased to 0.96. We initially identified 151,232 unique peptides and 11,177 proteins. After applying these filters, we quantified 103,990 unique peptides and 10,351 proteins, representing a 54% and 20% improvement, respectively, compared to the data acquired on the Orbitrap Ascend with a 90-min HRMS2 method (Fig. 1*G*).

### TMT-Based DDA and Label-Free DIA

**Fig. 2:**
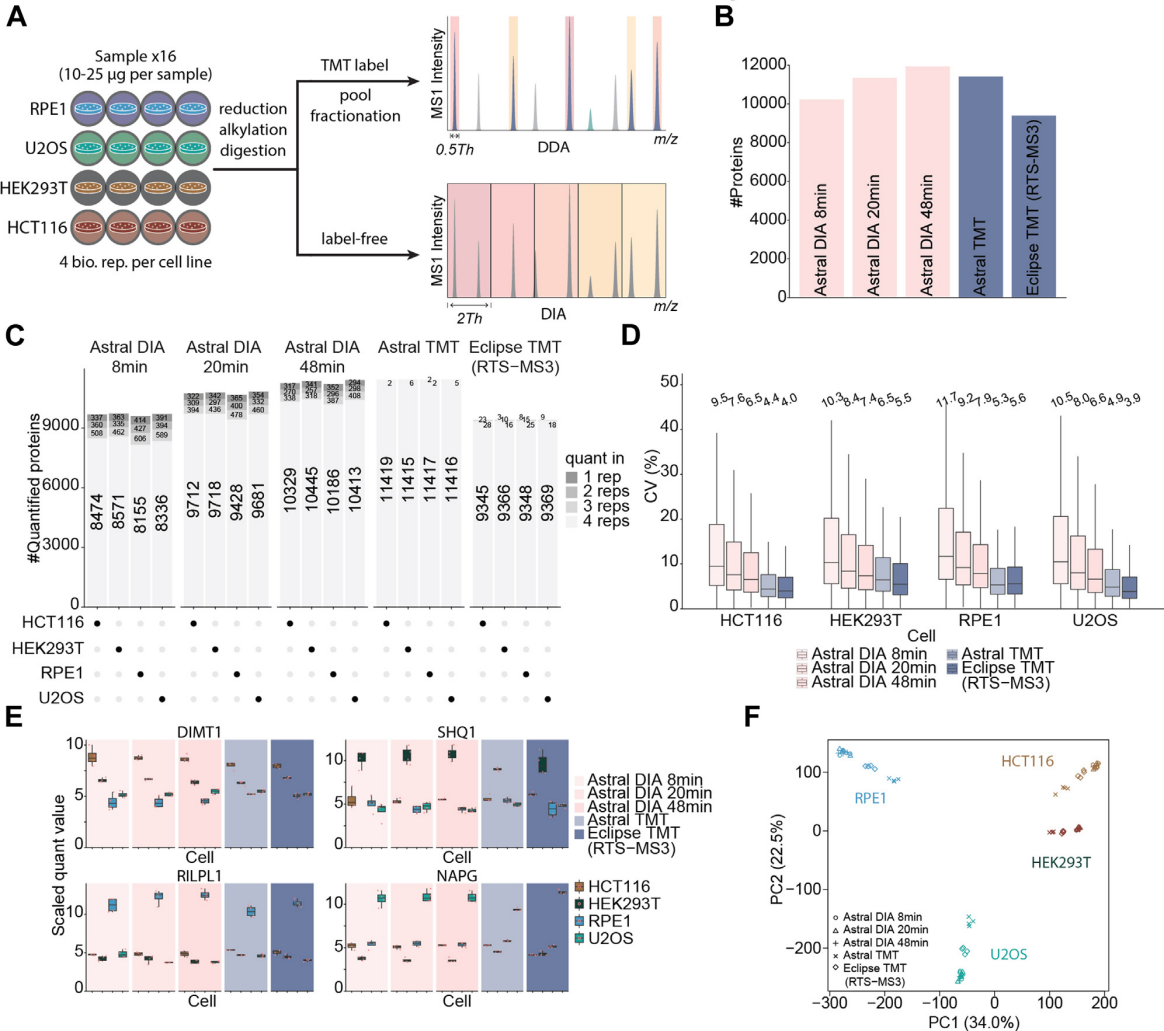
**Proteome-wide comparison between label-free DIA and TMT on the Orbitrap Astral mass spectrometer.***A*, diagram showing the experimental setup. Each cell line was cultured in four biological replicates. *B*, bar plot showing the total number of proteins identified with each method. *C*, proteins were stratified by the number of replicates in which they were quantified, considering only those quantified in at least one replicate. *D*, box plot showing the coefficient of variation (CV) of proteins among the DIA and TMT methods. The values above the boxes are the median CV. *E*, examples of protein quantification events. *F*, PCA plot showing the pattern of the 4 cell lines using the shared proteins. Astral, asymmetric track lossless; DIA, data-independent acquisition; PCA, principal component analysis; TMT, tandem mass tag.

Previous studies have demonstrated that the Orbitrap Astral significantly enhances the depth and throughput of proteomic analyses using a narrow-window DIA approach (8, 9, 10, 11, 12). However, a comparison between TMT and DIA on the Astral has not been published to our knowledge. We created a sample containing tryptic peptides from biological quadruplicates of four human cell lines (RPE1, U2OS, HEK293T, and HCT116) (Fig. 2*A* and supplemental Data S2). These peptides were TMT-labeled, pooled, and then fractionated. Twelve fractions were analyzed using a 90-min HRMS2 method on the Astral—equivalent to 67-min per sample. For comparison, the 16 unlabeled and unfractionated peptide samples were subjected to DIA analysis with three different gradient lengths—8, 20, and 48 min. As the Astral analyzer only supports HRMS2-based TMT quantification, which is susceptible to ratio compression (7), we decided to analyze the TMT-labeled sample using a 90-min real-time search-based MS3 method (RTS-MS3) on the Orbitrap Eclipse. We reasoned that RTS-TMT dataset could provide a more accurate quantification result as reference (7).

Across all cell line replicates, we identified 10,231, 11,345, 11,933, 11,421, and 9396 proteins using DIA 8 min, DIA 20 min, DIA 48 min, Astral TMT, and Eclipse TMT (RTS-MS3), respectively (Fig. 2*B*). While DIA-8 min achieved great proteome coverage, this dataset contained a substantial number of missing values, with fewer than 8600 proteins quantified in all quadruplicates of each cell line. With the DIA-48 min, approximately 800 proteins were missing in at least one replicate. In contrast, Astral TMT and Eclipse TMT (RTS-MS3) produced nearly complete datasets (Fig. 2*C*). In addition, we assessed the interplex data completeness by injecting the fractionated four-cell-line TMT samples twice on both the Astral and the Eclipse, where approximately 95% of proteins and 80% unique peptides were quantified in both replicates on either platform (supplemental Fig. S4).

Next, we evaluated the CV of protein measurements for the cell lines across all methods. DIA-8 min exhibited the highest CV (median ∼ 10%). Increasing gradient length effectively lowered CV values for DIA; however, they were still significantly higher than Astral TMT and Eclipse TMT (Fig. 2*D*). Eclipse TMT showed the lowest CV (median ∼4–5%), owing to its ability to reduce interference and improve accuracy. The trend was more obvious at the peptide level, with DIA-8 min presenting a median CV of over 20% compared to TMT’s ∼ 10% (supplemental Fig. S5*A*).

As illustrated in Figure 2*E*, we selected four example proteins that are highly abundant in each cell line. Differential abundance was observed across all methods. Combining the 8300 proteins shared across all methods into one dataset revealed differential abundances proteome-wide, as seen in the heatmap where cell lines are hierarchically clustered (supplemental Fig. S6). At the peptide level, 53,208 peptide sequences were still shared across all five methods (supplemental Fig. S5*B*). Interestingly, principal component analysis of the shared 8300 proteins quantified across all datasets showed that, although the main driver is the cell line, differences between cell lines were "compressed" in Astral TMT data, while Astral DIA data showed more separation (Fig. 2*F*, supplemental Figs. S5*C*, and S7). We attributed the observation to ratio compression with TMT data, which will be explored further in the following section.

Inspired by the work of Wen *et al.* (27) which suggests suboptimal FDR control in DIA data processing, we evaluated the FDR in our DIA dataset and arrived at a similar conclusion (supplemental Fig. S8). Specifically at the protein level, when the FDR threshold was set to 1%, the estimated FDR for DIA exceeded 3%, whereas it remained below 1% for TMT.

### Evaluation of Quantitative Accuracy Using a Two-Proteome Interference Model

**Fig. 3:**
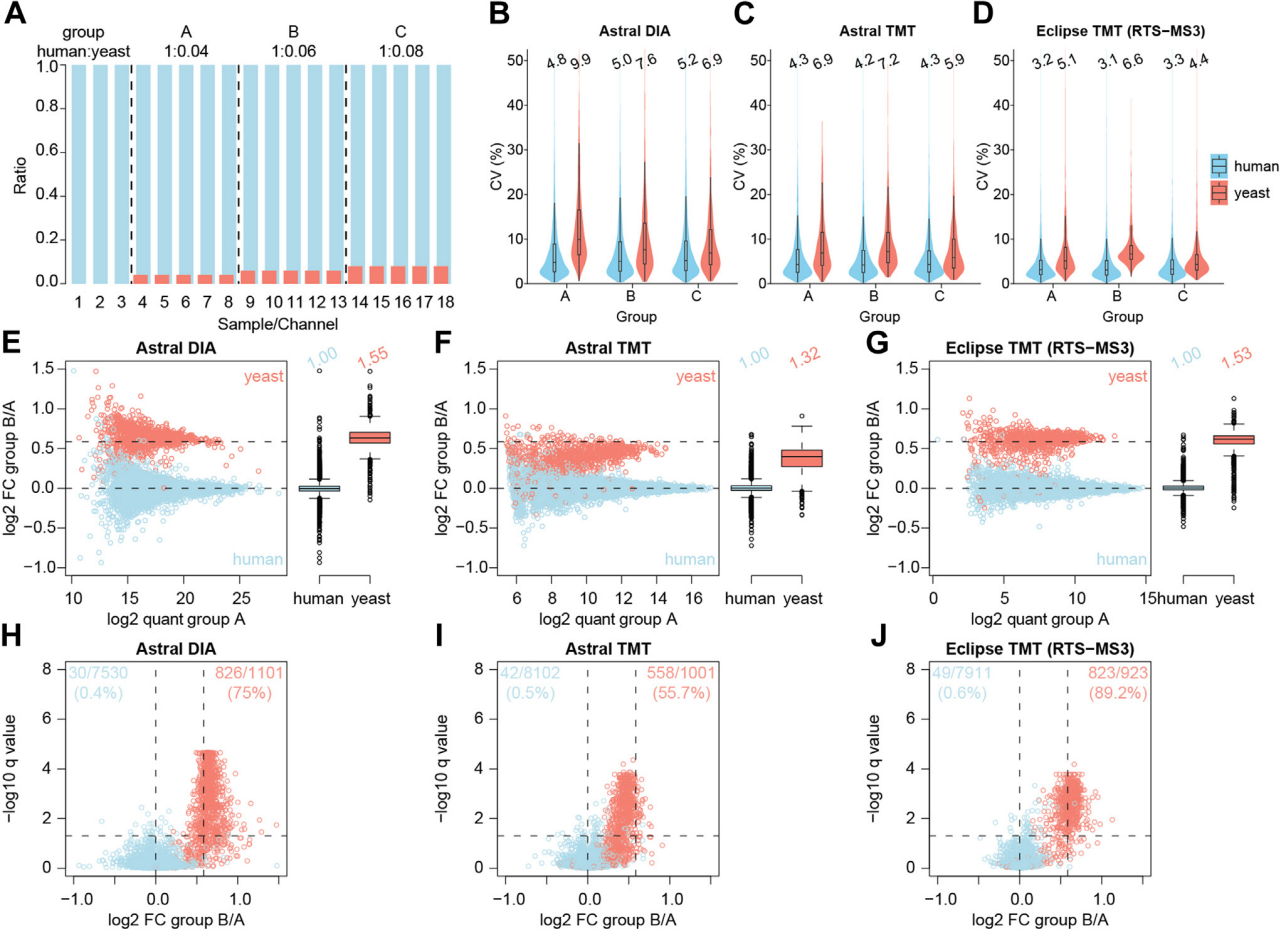
**Assessing the quantitative precision and accuracy for DIA and TMT on the Orbitrap Astral.***A*, bar plot showing the experiment setup. *B–D*, Violin plots showing CVs of (*B*) DIA by the Orbitrap Astral, (*C*) TMT by the Orbitrap Astral, and (*D*) TMT by the Orbitrap Eclipse using the RTS-MS3 method. *E*–*G*, scatter plots with box plots showing measured fold changes for (*E*) DIA by the Orbitrap Astral, (*F*) TMT by the Orbitrap Astral, and (*G*) TMT by the Orbitrap Eclipse using RTS-MS3 method, when the theoretical fold change is 1.5. Volcano plots showing the numbers of significant proteins out of the total number of quantified proteins for (*H*) DIA by the Orbitrap Astral, (*I*) TMT by the Orbitrap Astral, and (*J*) TMT by the Orbitrap Eclipse using RTS-MS3 method, when the theoretical fold change is 1.5. Yeast proteins are colored in coral while human proteins are colored in *sky-blue*. Astral, asymmetric track lossless; CV, coefficient of variation; DIA, data-independent acquisition; RTS-MS3, real-time search-based MS3; TMT, tandem mass tag.

To benchmark the quantitative performance of the TMT-based approach on the Orbitrap Astral, we generated a two-proteome interference model consisting of human and yeast peptides at a 20:1 ratio (w/w; Fig. 3*A* and supplemental Data S3). The low abundance yeast peptides had variable ratios (1, 1.5, and 2) and was mixed with a substantial and consistent amount of human interference (17, 28). This setup is designed to understand the quantitative performance of low-abundance proteins. The TMT-labeled sample was fractionated into 12 fractions, and each fraction was analyzed using a 90 min method. We measured the deviation from the defined ratios as an indicator of interference from co-isolation, which tends to compress yeast peptide ratios toward 1:1. For comparison, the unlabeled and unfractionated peptide samples were subjected to 42 min DIA analysis on the Orbitrap Astral. The same TMT-labeled samples were analyzed using the Orbitrap Eclipse with the 90 min RTS-TMT method to recover accuracy.

We first examined the quantitative precision of DIA, TMT, and RTS-TMT for both human and yeast proteins across the three ratio groups (Fig. 3, *A*–*D*). As an example in ratio group A, DIA exhibited higher protein CVs, with a median of 9.9% for yeast proteins. In contrast, TMT produced a lower median CV of 6.9%. The RTS-TMT showed the highest precision, with a median protein CV of 5.1%. Quantification precision correlated with signal, with lower signal showing higher CV values (supplemental Figs. S9 and S10). Overall, TMT generated lower CVs compared to DIA, and RTS-TMT outperformed HRMS2-based TMT.

We assessed further the quantitative accuracy of Astral DIA, Astral TMT, and Eclipse TMT (RTS-MS3) for both human and yeast proteins by calculating the fold changes of group B *versus* group A and group C *versus* group A (Fig. 3, *E*–*G* and supplemental Fig. S11, *A*–*C*). Figure 3*, E–G* present scatter plots of the log2 ratios *versus* quantification for human (sky blue) and yeast (coral) proteins, with theoretical ratios of 1 and 1.5 indicated by horizontal dashed lines. The box plots to the right of each scatter plot illustrate the distribution of log2 ratios. supplemental Fig. S11, *A*–*C* show the measured ratios when theoretical ratios are 1 and 2 for human and yeast proteins. Both DIA and RTS-TMT demonstrated high accuracy. However, we observed ratio compression with Astral TMT, due to ion interference. In addition to acquiring MS2 in the Astral analyzer, we collected MS2 in the Orbitrap and observed similar ratio compression (supplemental Fig. S12). Similar to precision, accuracy fluctuated more dramatically with lower signal measurements (Fig. 3, *E*–*G* and supplemental Fig. S11, *A*–*C*). The ratio compression in turn negatively affected statistical significance. Theoretically, all yeast proteins are expected to exhibit significant differences between group B and group A (1.5-fold changes). However, our analysis revealed that 75% of yeast proteins were significantly different in measured abundance (q < 0.05) using Astral DIA, 55.7% using Astral TMT, and 89.2% using Eclipse RTS-TMT (Fig. 3, *H–J*). For group C *versus* group A (2-fold changes), the percentages of significant yeast proteins identified were 90%, 78.3%, and 95.5%, respectively, across the three methods (supplemental Fig. S11, *D*–*F*). Notably, for low-abundance proteins subjected to substantial ion interference, RTS-TMT demonstrated better sensitivity in detecting 1.5- and 2-fold changes. We conclude that while Astral DIA exhibits great quantitative accuracy overall, Astral TMT was negatively affected by ratio compression, which is inherent to MS2-based method (7). We anticipate that integrating MS3 capability or using a more advanced quadrupole that allows for narrower isolation would enhance the performance of TMT on the Orbitrap Astral.

### TMT-Based Reactive Cysteine Profiling

**Fig. 4:**
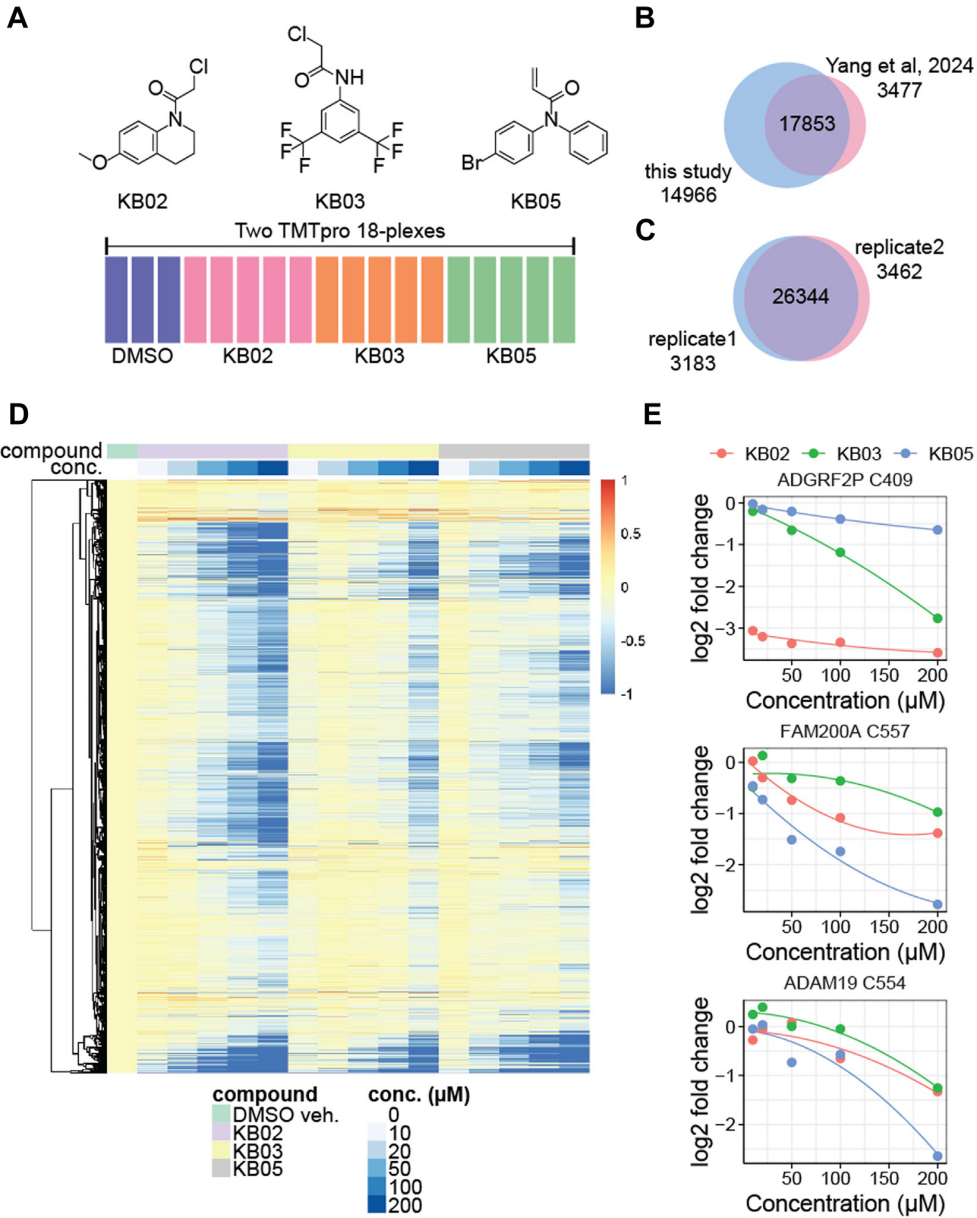
**Benchmarking TMT-based reactive cysteine profiling on the Astral.***A*, diagram showing the experimental setup. *B* and *C*, Venn diagrams showing number of unique localized desthiobiotin iodoacetamide-cysteine sites (*B*) between this study and our previous results on the Orbitrap Eclipse and (*C*) between two replicates. *D*, heatmap of log2-fold changes across all cysteines quantified. *E*, examples of newly identified and ligandable cysteines that were not identified in previously published datasets profiling the fragments. Astral, asymmetric track lossless; TMT, tandem mass tag.

Prompted by the observation that TMT significantly improves depth with quantitative precision at the peptide level on the Astral, we further assessed its utility in ABPP of reactive cysteine residues. ABPP has become a powerful approach in probing protein function and identifying tractable binding pockets (15, 16, 29, 30). In our previous studies, we developed a reactive cysteine profiling technique, TMT-ABPP, which combines a desthiobiotin iodoacetamide probe, TMT-multiplexing, and advanced mass spectrometry technologies for high-throughput and global profiling of reactive cysteines (15, 16). To benchmark TMT-based reactive cysteine profiling on the Astral, we treated 10 μg of HEK293T cell lysates with varying concentrations (0–200 μM) of three well-characterized, highly reactive scout fragments—KB02, KB03, and KB05 (29)—for 1 h (Fig. 4*A* and supplemental Data S4). Technical duplicates were generated. In single-shot 3-h LC-MS analyses, we identified and quantified nearly 33,000 unique cysteines across 18 samples (Fig. 4*B*), representing a ∼54% increase compared to our previous results on the Orbitrap Eclipse mass spectrometer (15). Of the unique cysteines identified, 26,344 (∼90%) were detected in both replicates (Fig. 4*C*. The dose-dependent fold changes observed in thousands of cysteines treated with the three fragments further corroborated their broad reactivity (Fig. 4*D*).

These data enabled an in-depth examination of cysteine ligandability. We discovered previously unknown ligandable cysteines (Fig. 4*E*). These newly identified reactive cysteines exhibited distinct ligandability profiles across the three fragments, highlighting the combined influence of the warhead and binding groups on selectivity. For example, KB02 emerged as the most potent binder of C409 on the adhesion G protein-coupled receptor F2P (gene name: ADGRF2P), while KB05 preferentially targeted C557 of FAM200A and C554 of ADAM19 (Fig. 4*E*). The ability to access more cysteines and uncover new ligandable sites underscores the sensitivity of TMT-ABPP on the Astral.

### KRAS G12C Covalent Drugs Show Off-Target Effects

**Fig. 5:**
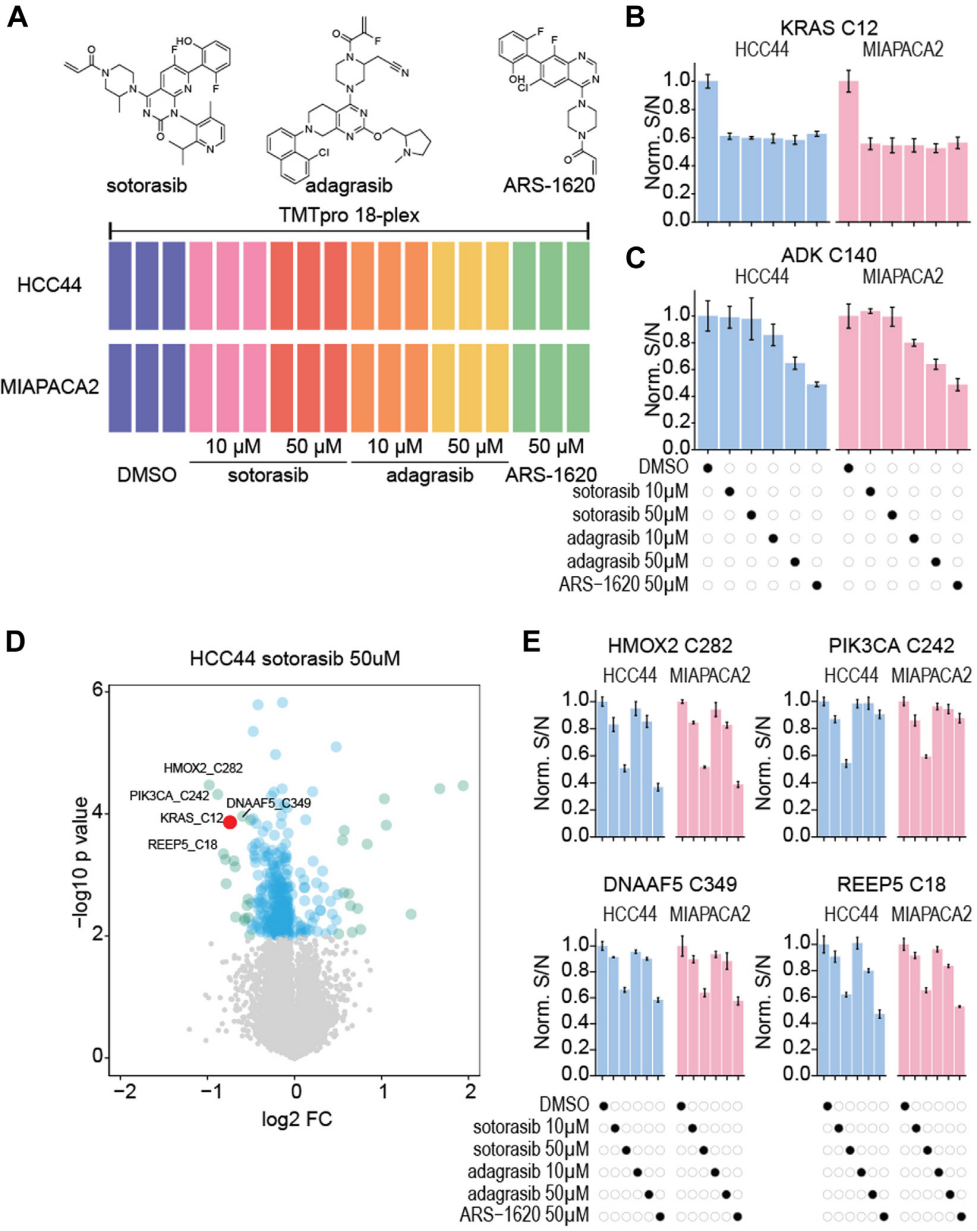
**Assessing off-targets of KRAS G12C covalent drugs by TMT-based ABPP on the Astral.***A*, diagram showing the experimental setup. *B*, bar plots showing engagement of KRAS C12 with KRAS G12C covalent drugs. *C*, bar plots showing engagement of ADK C140 with KRAS G12C covalent drugs. *D*, Volcano plot showing significant engagement of cysteine sites with sotorasib in HCC44. *E*, bar plots showing engagement of significant cysteine sites in (*D*) with KRAS G12C covalent drugs. ABPP, activity-based proteome profiling; Astral, asymmetric track lossless; TMT, tandem mass tag.

We demonstrated that TMT-ABPP works effectively on the Orbitrap Astral mass spectrometer. Building on this observation, we applied it to investigate potential unknown off-target effects of well-characterized KRAS G12C covalent inhibitors: sotorasib (31), adagrasib (32), and ARS-1620 (33) (Fig. 5*A* and supplemental Data S5). The KRAS G12C mutation locks KRAS in a GTP-bound active form, activating downstream oncogenic pathways and promoting tumorigenesis. To assess drug engagement, we conducted experiments using native cell lysates from two KRAS G12C cell lines: HCC44 (lung adenocarcinoma) and MIAPACA2 (pancreatic adenocarcinoma). We treated 20 μg of cell lysate with each compound at concentrations of 10 μM and/or 50 μM for 1 h. KRAS C12 was significantly engaged with all three drugs in both cell lines (Fig. 5*B*). In addition, consistent with our recent report (15), ADK C140, a cysteine located within the active site of adenosine kinase, showed significant engagement with ARS-1620 in both cell lines (Fig. 5*C*). Interestingly, ADK C140 also exhibited dose-dependent engagement to adagrasib.

Although sotorasib and adagrasib primarily target KRAS C12, we observed significant engagement of off-target cysteine residues in other proteins at the concentration we tested (Fig. 5, *D*–*E* and supplemental Fig. S13). For instance, in sotorasib-treated samples, HMOX C282, PIK3CA C242, DNAAF5 C349, and REEP5 C18 emerged as top hits, while in adagrasib-treated samples, KNTC1 C1825, PLIN3 C341, and FECH C236 and C395 were prominently engaged. Many of these cysteines displayed dose dependency. Both sotorasib and adagrasib fully engaged all available KRAS C12 at the lowest concertation we tested (10 μM). To further evaluate the does-response relationship, we performed another experiment using a larger concentration range of the two molecules from 5 μM to 12.8 pM. Both inhibitors were highly potent with low nanomolar half-maximal target engagement values (supplemental Fig. S14), and most of the off targets no longer showed significant difference as expected. While the pharmacological effects arising from the off-targets merit further investigation, these findings highlight our TMT-ABPP approach in uncovering targets of covalent inhibitors. We anticipate this method to be broadly applicable to the field of chemoproteomics.

## Conclusion

Here we benchmarked the performance of the Orbitrap Astral mass spectrometer in the context of TMT-based multiplexed quantitative proteomics. We fully evaluated its merits and compared it with label-free DIA on the Orbitrap Astral and RTS-MS3 on the Orbitrap Eclipse. We presented a multidimensional assessment of proteome coverage, data completeness, quantitative precision, and accuracy across these methodologies.

In our experiments, the Orbitrap Astral demonstrated robust performance across both DIA- and TMT-based methods, each with its own strengths and limitations. We identified over 10,000 proteins in 16 proteomes within a day by TMT, comparable to the proteome coverage by DIA. However, using an orthogonal paired entrapment method, we found that the estimated FDR for DIA exceeded 3%, while TMT maintained an FDR below 1% when applying a standard 1% protein-level threshold (27). We observed that the Astral DIA method, while providing high quantitative accuracy and extensive proteome coverage, exhibited higher CVs especially for low-abundance proteins and peptides compared to TMT. The TMT-based method produced datasets with almost no missing values and lower CVs, although it was susceptible to ratio compression, which is inherent to MS2-based TMT methods. The Eclipse TMT (RTS-MS3) method, by contrast, consistently demonstrated the highest precision and improved accuracy.

Cysteine residues serve essential roles in protein function and cellular homeostasis (34). Targeting functional cysteines using electrophilic molecules has proven to be a promising strategy for addressing traditionally undruggable protein targets such as KRAS^G12C^ (35). TMT-ABPP stands out as a mainstream method for globally profiling covalently ligandable cysteines (36). We demonstrated that TMT-ABPP on the Astral improves the depth by 54% compared to the Orbitrap Eclipse and reveals off-targets of well-studied compounds including sotorasib and adagrasib.

The Orbitrap Astral mass spectrometer platform, with its advanced capabilities, is well-positioned to drive the next generation of proteomics research, enabling deeper insights into complex biological systems and facilitating the discovery of new biomarkers and therapeutic targets. The integration of TMTpro 35-plex (6) will further increase sample multiplexing and throughput, leveraging the Astral's high speed and resolution to accelerate proteomic analyses. We envision an improved TMT quantitative accuracy on the Orbitrap Astral, potentially through the integration of RTS-MS3 capabilities (7, 37, 38, 39) or enhanced quadrupole performance to mitigate ratio compression. This will be especially valuable for measuring low-abundance proteins.

## Data availability

Raw data are available in MassIVE repository (MSV000096674).

## Supplemental data

This article contains supplemental data.

## Conflict of interest

S. P. G. is on the advisory board for Thermo Fisher Scientific, Cedilla Therapeutics, Casma Therapeutics, Cell Signaling Technology, and Frontier Medicines. Q. Y. is a consultant and/or collaborator with Thermo Fisher Scientific, Vertex Pharmaceuticals, FL94 Inc, and Matchpoint Therapeutics. M. Z., G. C. M., H. I. S., C. H., E. D., and V. Z. are employees of Thermo Fisher Scientific, the manufacturer of the Orbitrap Astral mass spectrometer. Other authors declare no competing interests.
