# Supplementary material for: TMT-Based Multiplexed (Chemo)Proteomics on the Orbitrap Astral Mass Spectrometer
Source: Mol Cell Proteomics. 2025 Apr 8;24(5):100968. doi: 10.1016/j.mcpro.2025.100968 (PMC12127577; doi:10.1016/j.mcpro.2025.100968)
Supplement: Supplemental data [file mmc6.docx]

**TMT-based Multiplexed (Chemo)proteomics on the Orbitrap Astral Mass Spectrometer**

*Yuchen He^1^, Ka Yang^1^,* *Shaoxian Li^2^, Martin Zeller^3^, Graeme C. McAlister^4^, Hamish I. Stewart^3^, Christian Hock^3^, Eugen Damoc^3^, Vlad Zabrouskov^4^, Steven P. Gygi^1^, Joao A. Paulo^1,^*, and Qing Yu^2,^**

^1^ Department of Cell Biology, Harvard Medical School, Boston, MA 02115, United States.

^2^ Department of Biochemistry and Molecular Biotechnology, University of Massachusetts Chan Medical School, Worcester, MA 01605, United States

^3^ Thermo Fisher Scientific, Hanna-Kunath-Straße 11, 28199 Bremen, Germany.

^4^ Thermo Fisher Scientific, San Jose, CA 95134, United States.

* Correspondence: J.A.P. (joao_paulo@hms.harvard.edu) and Q.Y. ([qing.yu@umassmed.edu](mailto:qing.yu@umassmed.edu))

Table of Contents

[Supplemental Note 1. Determining resolution filter for Astral TMT data. S1](#_Toc194413991)

[Supplemental Figure 1. Assessing resolution and Astral MS2 AGC target using unfractionated TKO peptides. S2](#_Toc194413992)

[Supplemental Figure 2. Assessing #PSMs and TKO peptide amount on the Astral. S3](#_Toc194413993)

[Supplemental Figure 3. Assessing CVs of PSM against S/N. S4](#_Toc194413994)

[Supplemental Figure 4. Assessing the inter-plex TMT data completeness. S5](#_Toc194413995)

[Supplemental Figure 5. Peptide level comparison between label-free DIA and TMT data. S6](#_Toc194413996)

[Supplemental Figure 6. Overview of the proteomic data across five methods. S7](#_Toc194413997)

[Supplemental Figure 7. Correlation of fold changes between methods. S8](#_Toc194413998)

[Supplemental Figure 8. FDR evaluation. S9](#_Toc194413999)

[Supplemental Figure 9. Examination of protein CV for quartiles of quantification values. S10](#_Toc194414000)

[Supplemental Figure 10. Examination of peptide CV. S11](#_Toc194414001)

[Supplemental Figure 11. Additional assessment of the quantitative accuracy for DIA and TMT. S12](#_Toc194414002)

[Supplemental Figure 12. Additional assessment of the quantitative accuracy for TMT using Orbitrap for MS2. S13](#_Toc194414003)

[Supplemental Figure 13. Additional assessment of off-targets of KRAS G12C inhibitors by TMT-ABPP on the Astral. S14](#_Toc194414004)

[Supplemental Figure 14. Dose response of KRAS C12 to sotorasib and adagrasib. S15](#_Toc194414005)

Supplemental Note 1. Determining resolution filter for Astral TMT data.

For the resolution filter, assuming Gaussian-shaped mass spectral peaks under minimal space charge effects (Stewart et al., *J Mass Spectrom* 2024), the intensity $I$ can be expressed as:

$$I(m)= I_{0}e^{- \frac{{(m-m_{0})}^{2}}{{2\sigma}^{2}}}$$

where $I(m)$ is the intensity at position $m$, $I_{0}$ is the max intensity at the peak’s center $m_{0}$, and $\sigma$ is the standard deviation.

By $I\left( m \right)=$ $\frac{I_{0}}{2}$, we derive the $FWHM$ (full width at half maximum) as:

$$FWHM=2\sqrt{2ln2}\sigma$$

To achieve 10% baseline separation between two adjacent TMTpro reporter ion peaks (i.e. the n and c pair), the combined intensity at the valley midpoint must equal 10% of $I_{0}$. For two identical Gaussian peaks separated by $\Delta m$, this condition is:

$$2I_{0}e^{- \frac{{(\frac{\Delta m}{2})}^{2}}{{2\sigma}^{2}}}=10\%I_{0}$$

Solving for $\Delta m$

$$\Delta m\approx2.08FWHM$$

Then the required resolution is:

$$R=2.08\frac{m}{\Delta m}$$

For the TMTpro 134n/c reporter ion pair with a $\Delta m=0.00632 Da$:

$$R=2.08\times\frac{134}{0.00632}\approx44100 (rounded up to 45000)$$

Setting a resolution filter at 45000 for reporter ions with a > 200 S/N value produced improved quantification as shown in **Figure 1f**. For a 5% baseline separation, a resolution cutoff at approximately 49000 is needed the TMTpro 134n/c reporter ion pair.

**
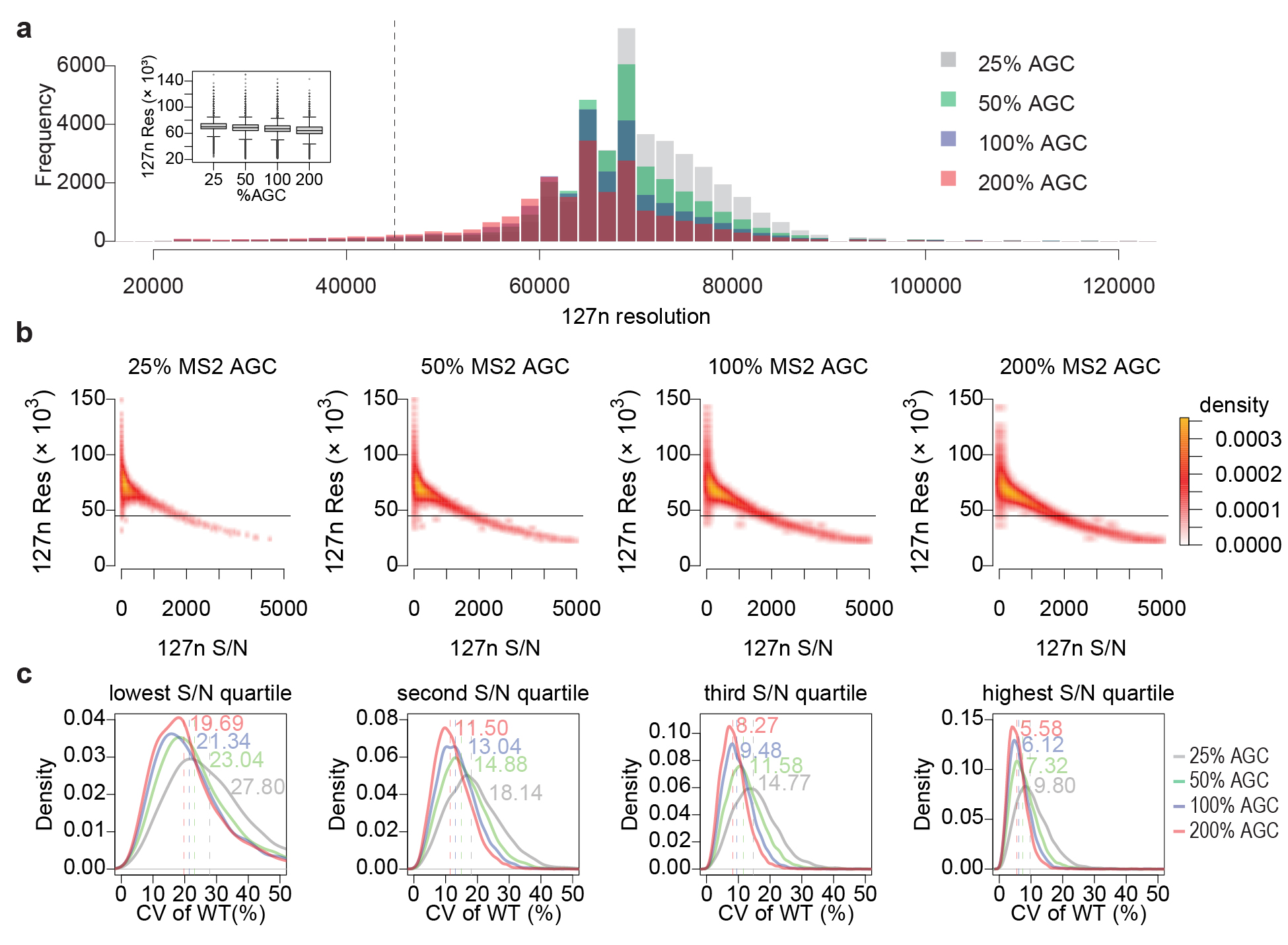
**

Supplemental Figure 1. Assessing resolution and Astral MS2 AGC target using unfractionated TKO peptides. TKO was made by TMTpro 16plex comprising S. cerevisiae wildtype, Δmet6, Δpfk2, and Δura2, each in four replicates. (a) Histogram and inset boxplot showing resolution of 127n reporter ion. (b) 2D density plots showing 127n reporter ion resolution against 127n signal-to-noise (S/N). (c) Density plots showing distributions of coefficient of variation (CV) of wildtype for different S/N quartiles. The numeric values in the plot are median CVs for each AGC target setting.

**
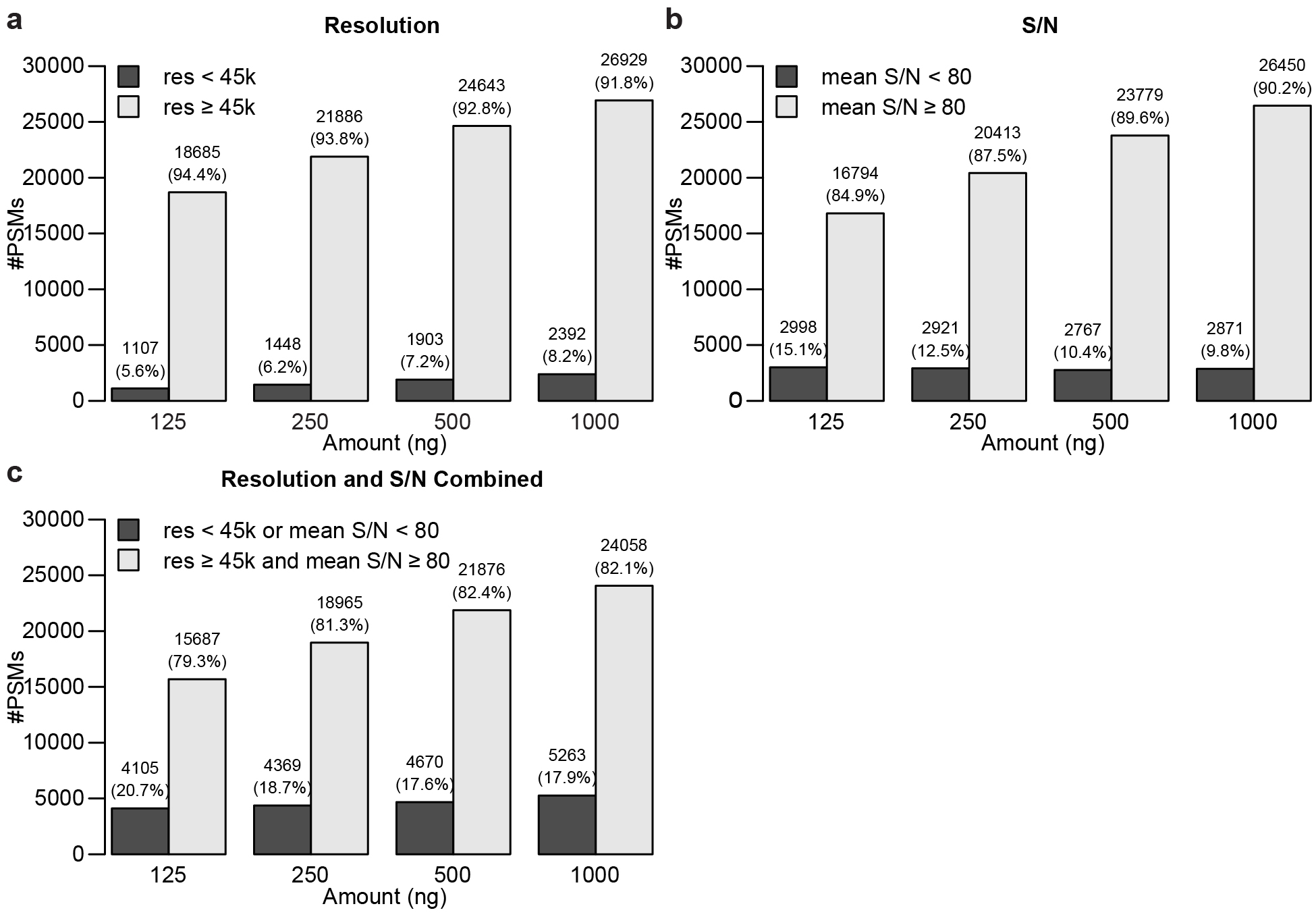
**

Supplemental Figure 2. Assessing #PSMs and TKO peptide amount on the Astral. (a) bar plot showing #PSMs categorized by resolution (≥ 45,000 vs. < 45,000) for reporter ions > 200 S/N. (b) bar plot showing #PSMs categorized by mean S/N (≥ 80 vs. < 80). (c) bar plot showing #PSMs passing (resolution ≥ 45,000 and mean S/N ≥ 80) and failing (resolution < 45,000 or mean S/N < 80) combined resolution and S/N thresholds.

**
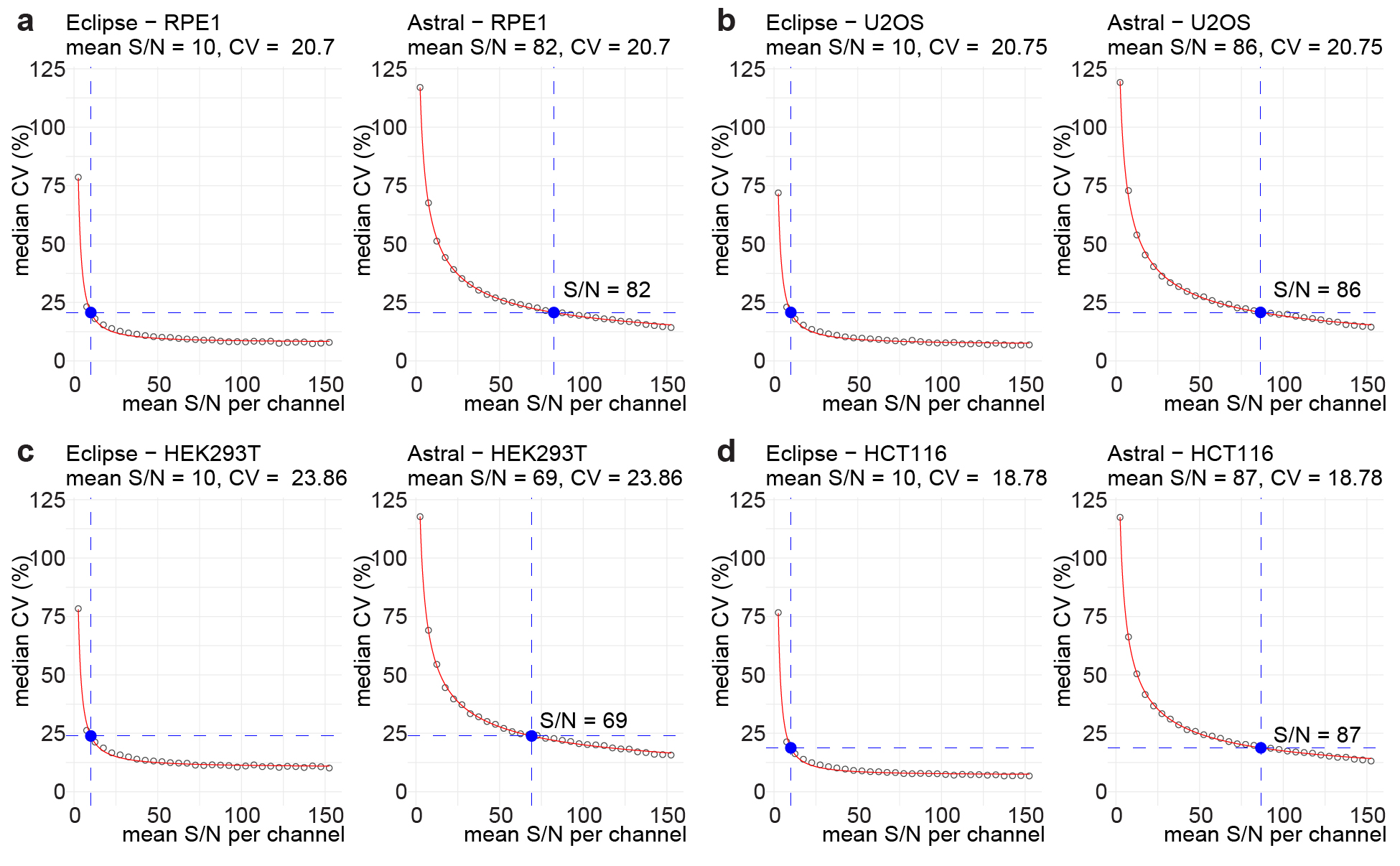
**

Supplemental Figure 3. Assessing CVs of PSM against S/N. The same fractionated TMT 16plex samples (four cell line, each in four replicates) were analyzed on the Eclipse and the Astral. For each cell line, the mean S/N and corresponding coefficient of variation (CV) were calculated per peptide. CV values were grouped into bins based on mean S/N ranges (5-S/N bins: 0–5, 5–10, 10–15, 15–20, etc.), and the median CV for each bin was plotted against the mean S/N for (a) RPE1, (b) USO2, (c) HEK293T, and (d) HCT116. In Orbitrap-based analyses, peptides are typically filtered to include only those with an average signal-to-noise (S/N) ratio above 10 per channel—a summed S/N of 180 for TMTpro 18-plex—to minimize high variability in peptide measurements (Li et al., *J. Proteome Res* 2021). Similarly, to achieve a CV for Astral-quantified peptides comparable to that of the Orbitrap, an average S/N filter of 80 per channel (summed S/N of 1440 for TMTpro 18-plex) is necessary.

**
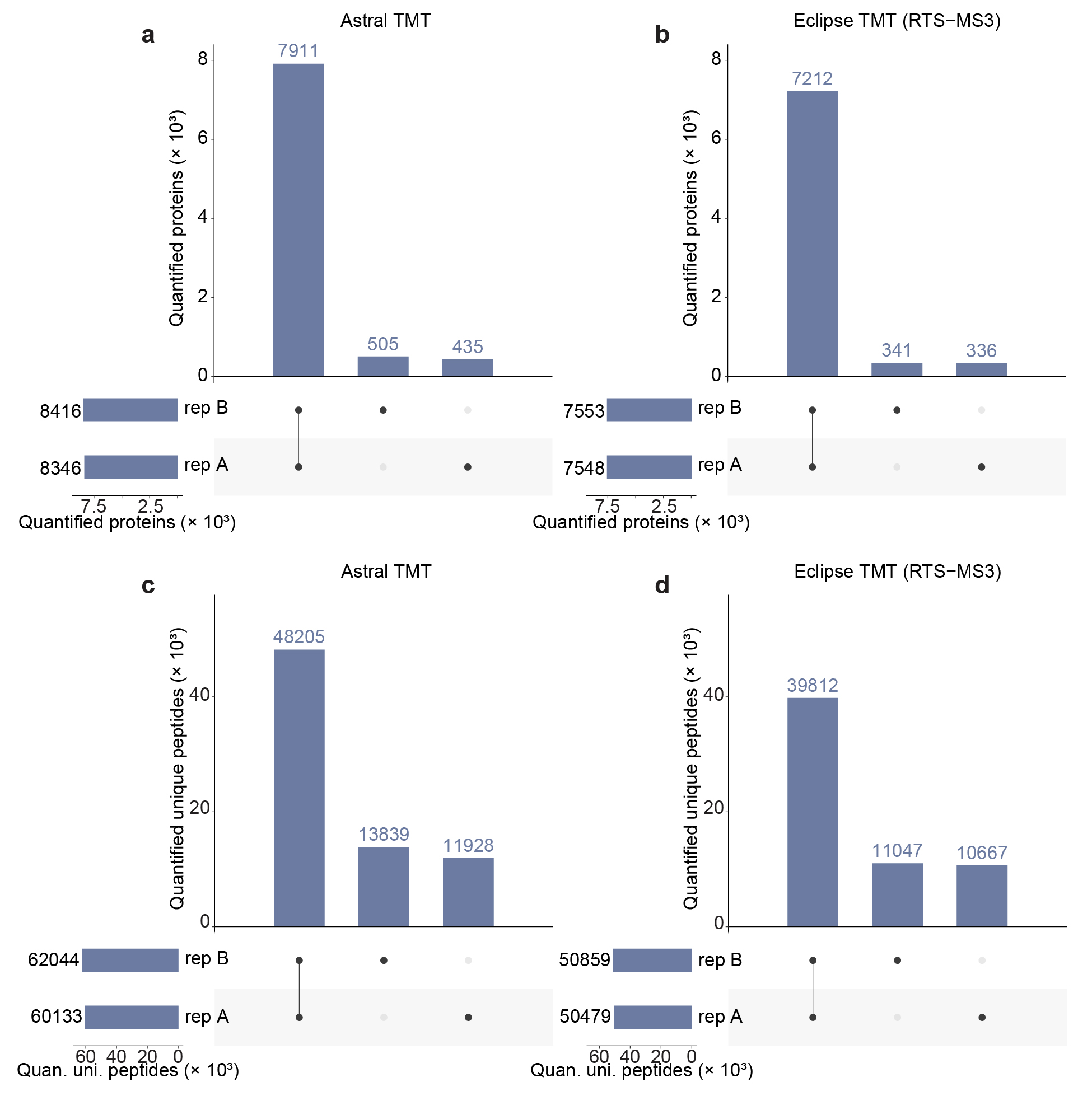
**

Supplemental Figure 4. Assessing the inter-plex TMT data completeness. The data collected on the Astral used 75 min gradient while the data collected on the Eclipse used 90 min gradient. The number of quantified proteins was shown in (a-b). The number of quantified unique peptides was shown in (c-d).

**
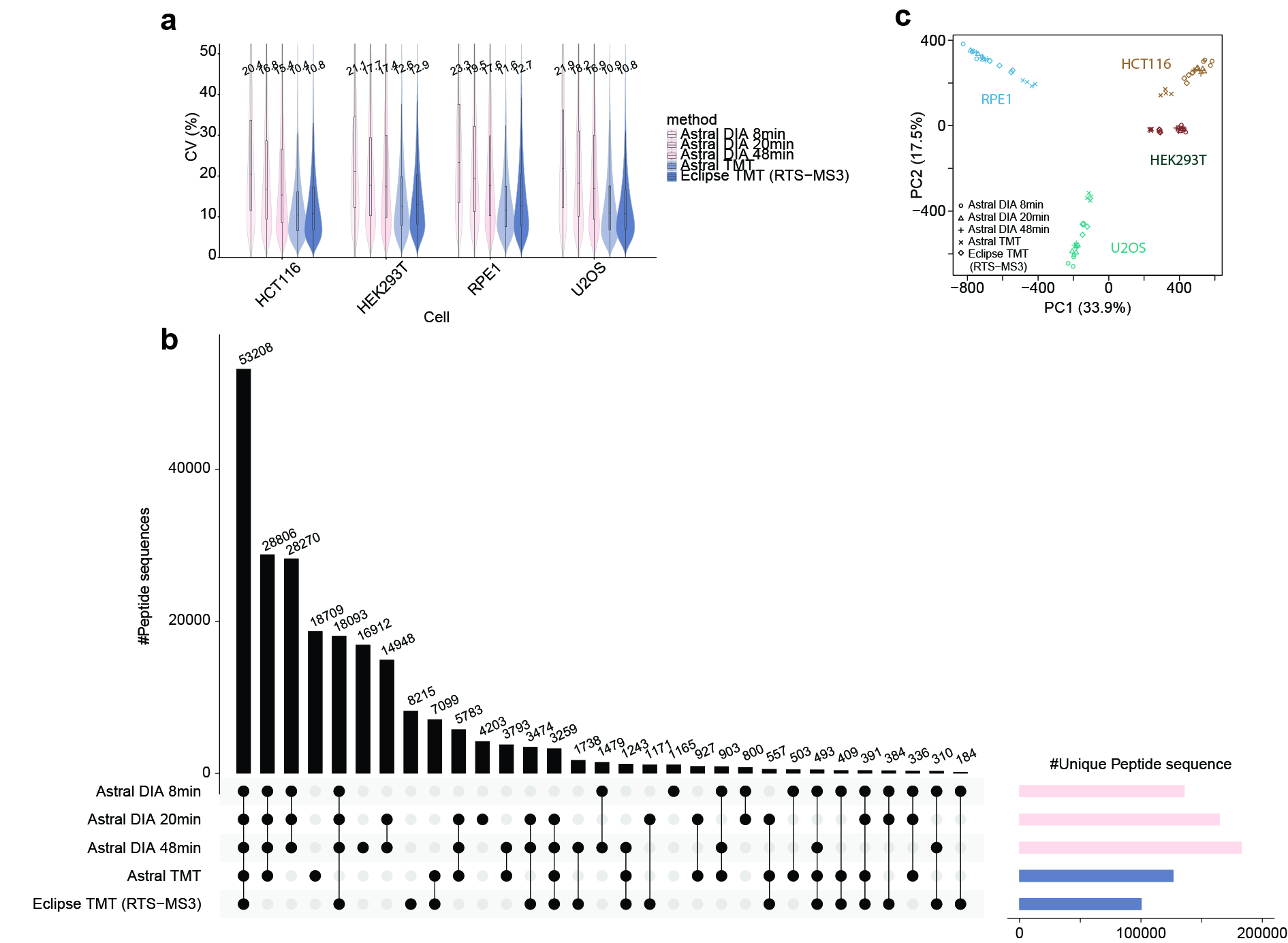
**

Supplemental Figure 5. Peptide level comparison between label-free DIA and TMT data. (a) Violin plot showing CVs of peptides among the DIA and TMT methods. The values above the violins are the median coefficient of variation. (b) Upset plot showing shared peptides among the DIA and TMT methods (c) PCA plot showing the pattern of the four cell lines using the shared peptides.

**
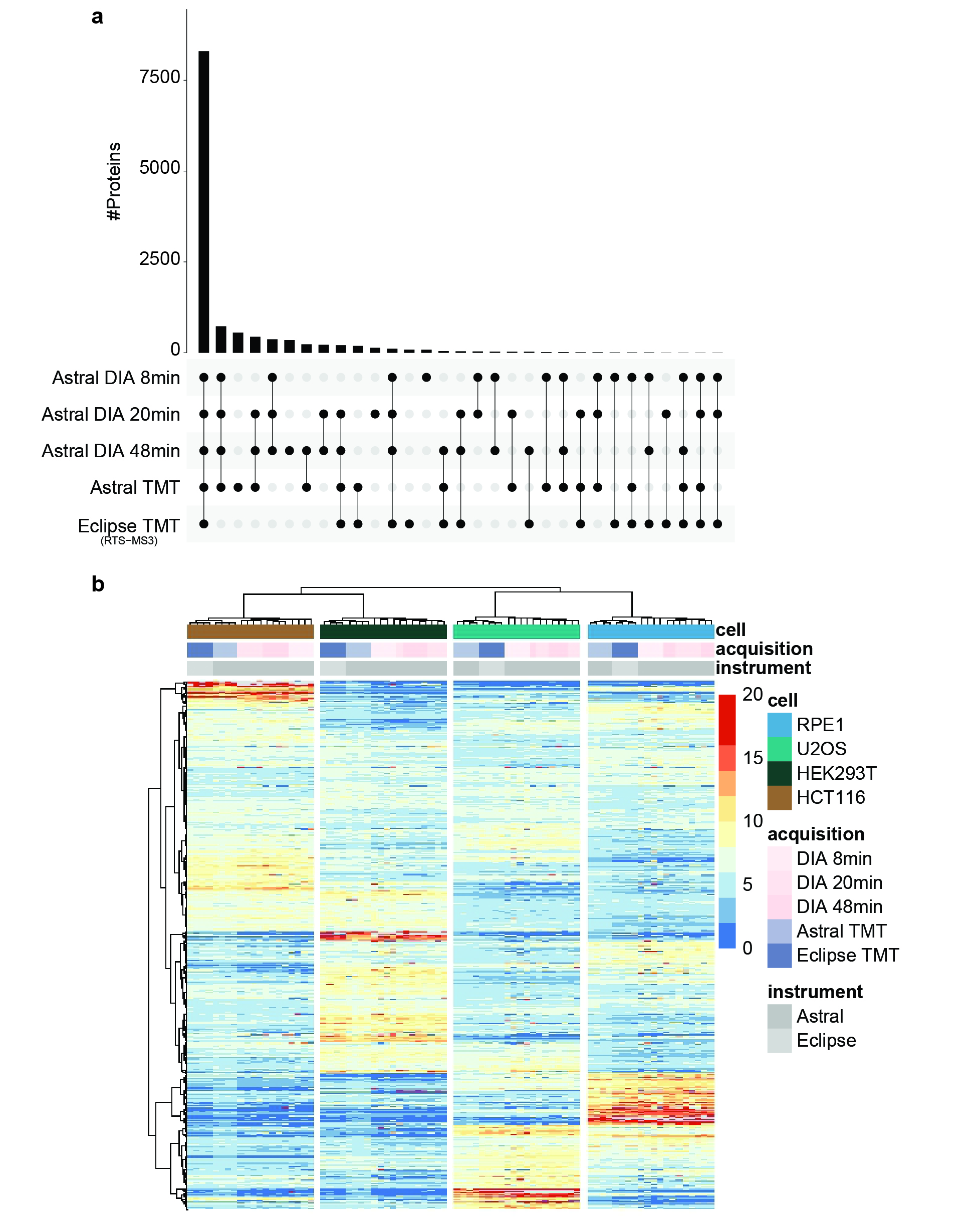
**

Supplemental Figure 6. Overview of the proteomic data across five methods. (a) Upset plot showing that the majority of proteins were detected across all five methods examined. (b) Heatmap showing differentially abundant proteins hierarchically clustered.

**
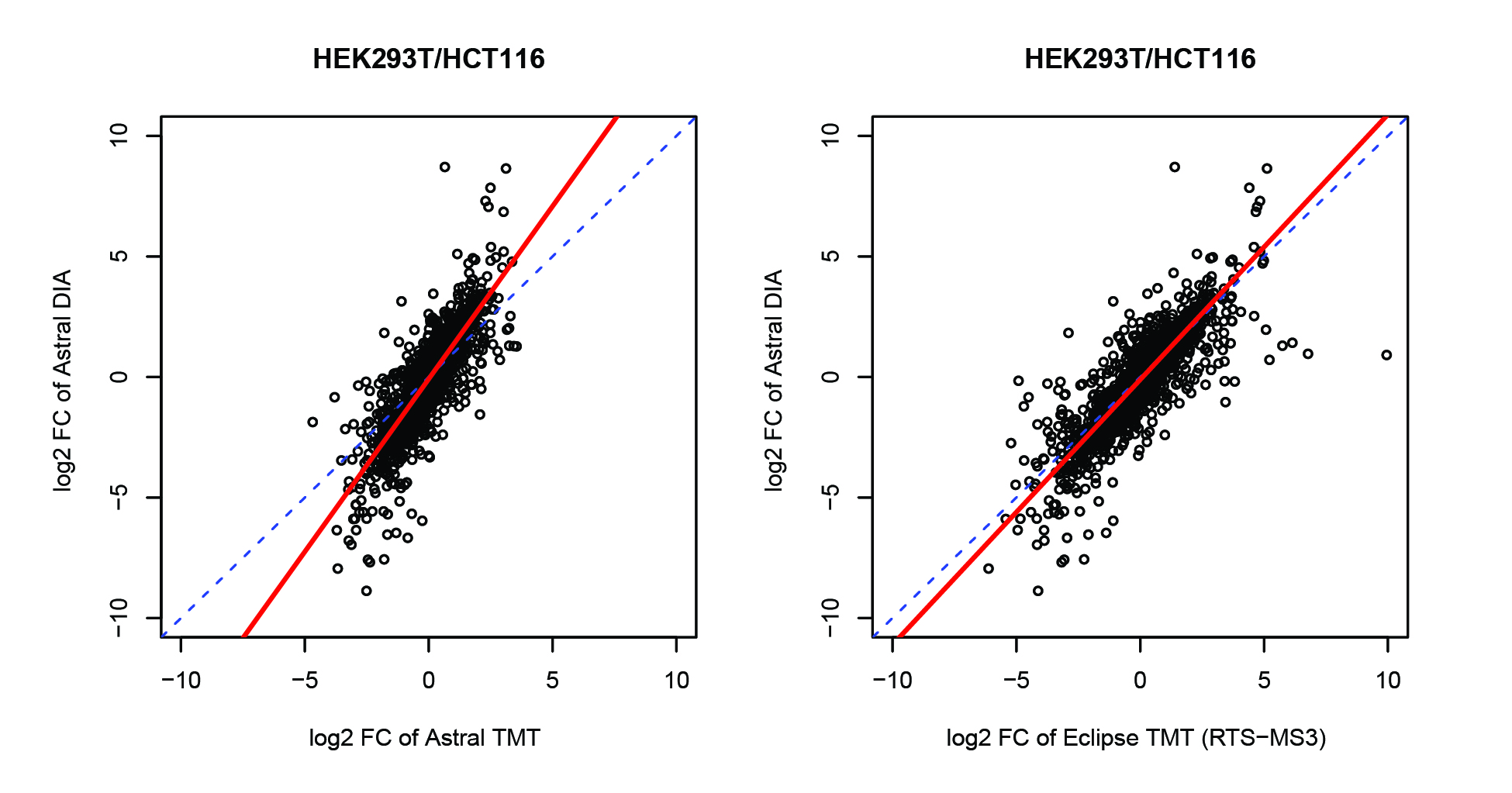
**

Supplemental Figure 7. Correlation of fold changes between methods. Data from Astral DIA 48 min and Astral/Eclipse TMT were compared. The dashed blue line is the diagonal line. The solid red line was generated using robust linear regression.

**
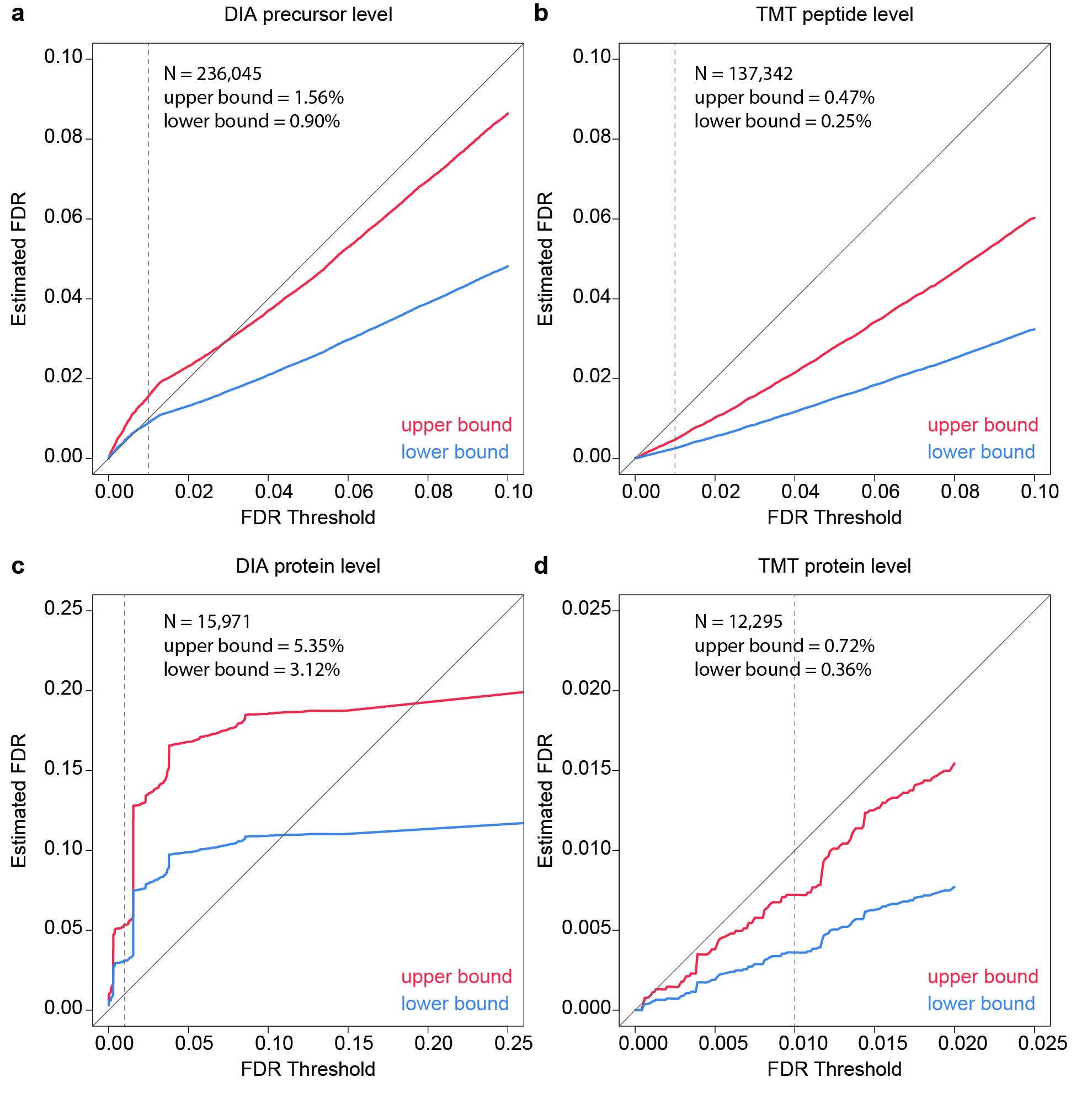
**

Supplemental Figure 8. FDR evaluation. Data from Astral DIA 48 min and Astral TMT were used to estimate the FDR at (a)-(b) precursor/peptide level and (c)-(d) protein level. DIA data was processed by DIA-NN v1.8.1. DDA data was processed by a Comet-based in-house built pipeline. The database used contains the original targets and the paired entrapment sequences. The vertical dashed grey line indicates the FDR threshold of 1%. The percentage values of upper and lower bound shown in each plot were the estimated FDR at the 1% FDR threshold.

**
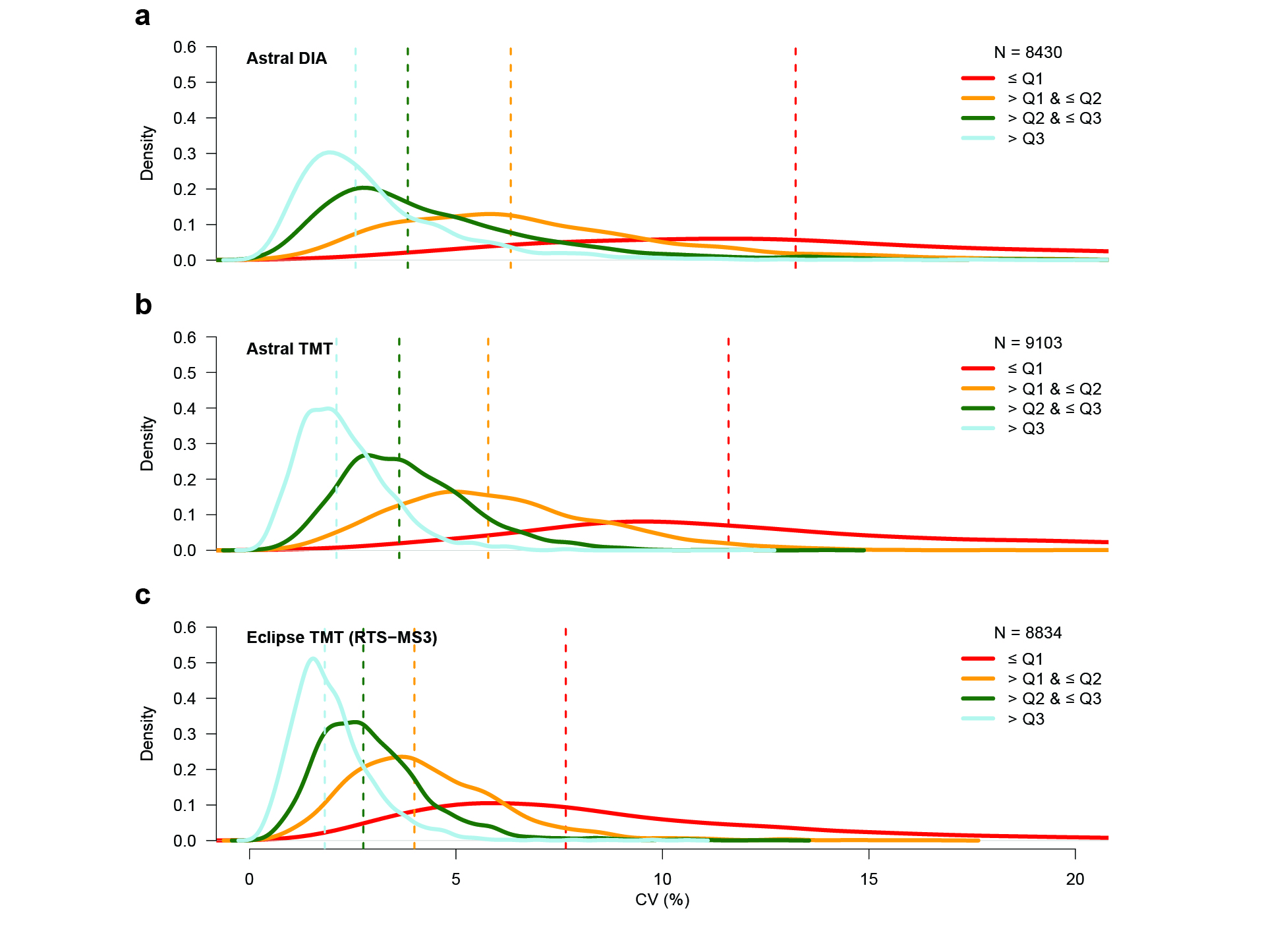
**

Supplemental Figure 9. Examination of protein CV for quartiles of quantification values. All proteins from group A were assigned into one of the four quartile based on their quantification values. The density plots show the distribution of protein CVs and the dashed vertical lines represent the median values for (a) DIA by the Orbitrap Astral, (c) TMT by the Orbitrap Astral, and (d) TMT by the Orbitrap Eclipse using RTS-MS3 method.


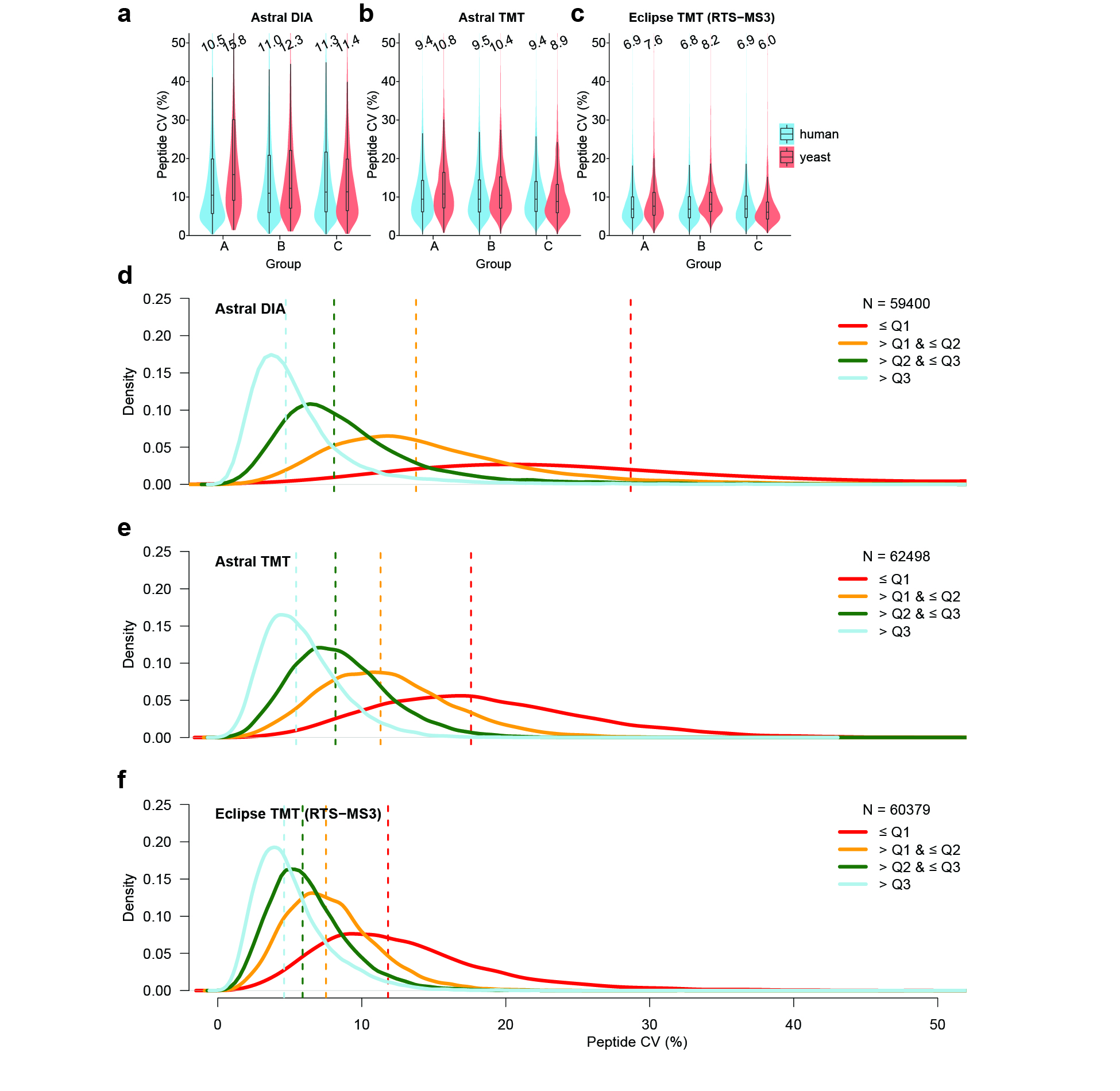


Supplemental Figure 10. Examination of peptide CV. Violin plots showing CV of (a) DIA by the Orbitrap Astral, (c) TMT by the Orbitrap Astral, and (d) TMT by the Orbitrap Eclipse using RTS-MS3 method. All peptides from group A were collapsed based on their sequence and were assigned into one of the four quartiles based on their quantification values. The density plots show the distribution of peptide CVs and the dashed vertical lines represent the median values for (d) DIA by the Orbitrap Astral, (e) TMT by the Orbitrap Astral, and (f) TMT by the Orbitrap Eclipse using RTS-MS3 method.


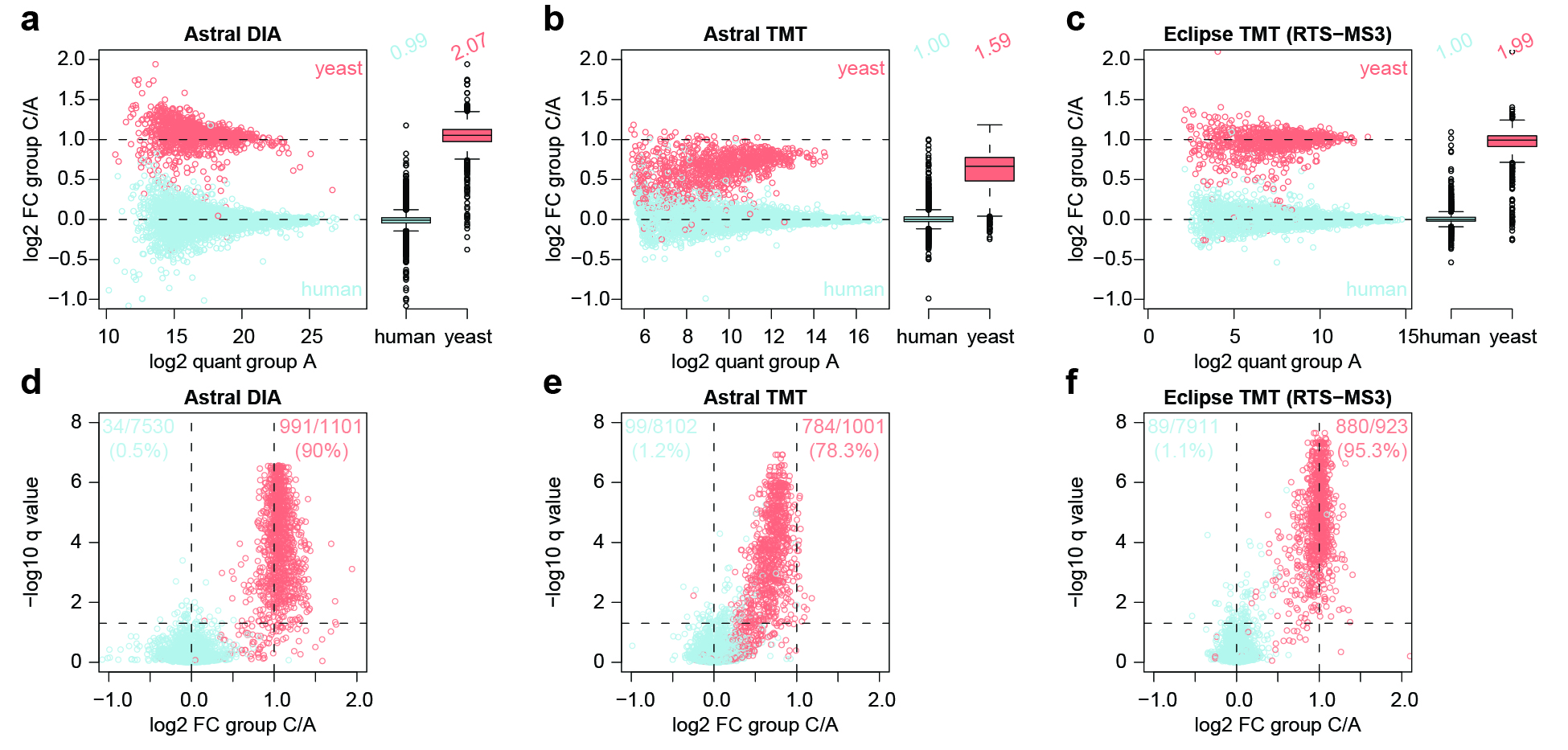


Supplemental Figure 11. Additional assessment of the quantitative accuracy for DIA and TMT. Scatter plots with boxplots showing measured fold changes for (a) DIA by the Orbitrap Astral, (b) TMT by the Orbitrap Astral, and (c) TMT by the Orbitrap Eclipse using RTS-MS3 method, when the theoretical fold change is 2. Volcano plots showing the numbers of significant proteins out of the total number of quantified proteins for (d) DIA by the Orbitrap Astral, (e) TMT by the Orbitrap Astral, and (f) TMT by the Orbitrap Eclipse using RTS-MS3 method, when the theoretical fold change is 2. Yeast proteins are colored in coral while human proteins are colored in sky-blue.


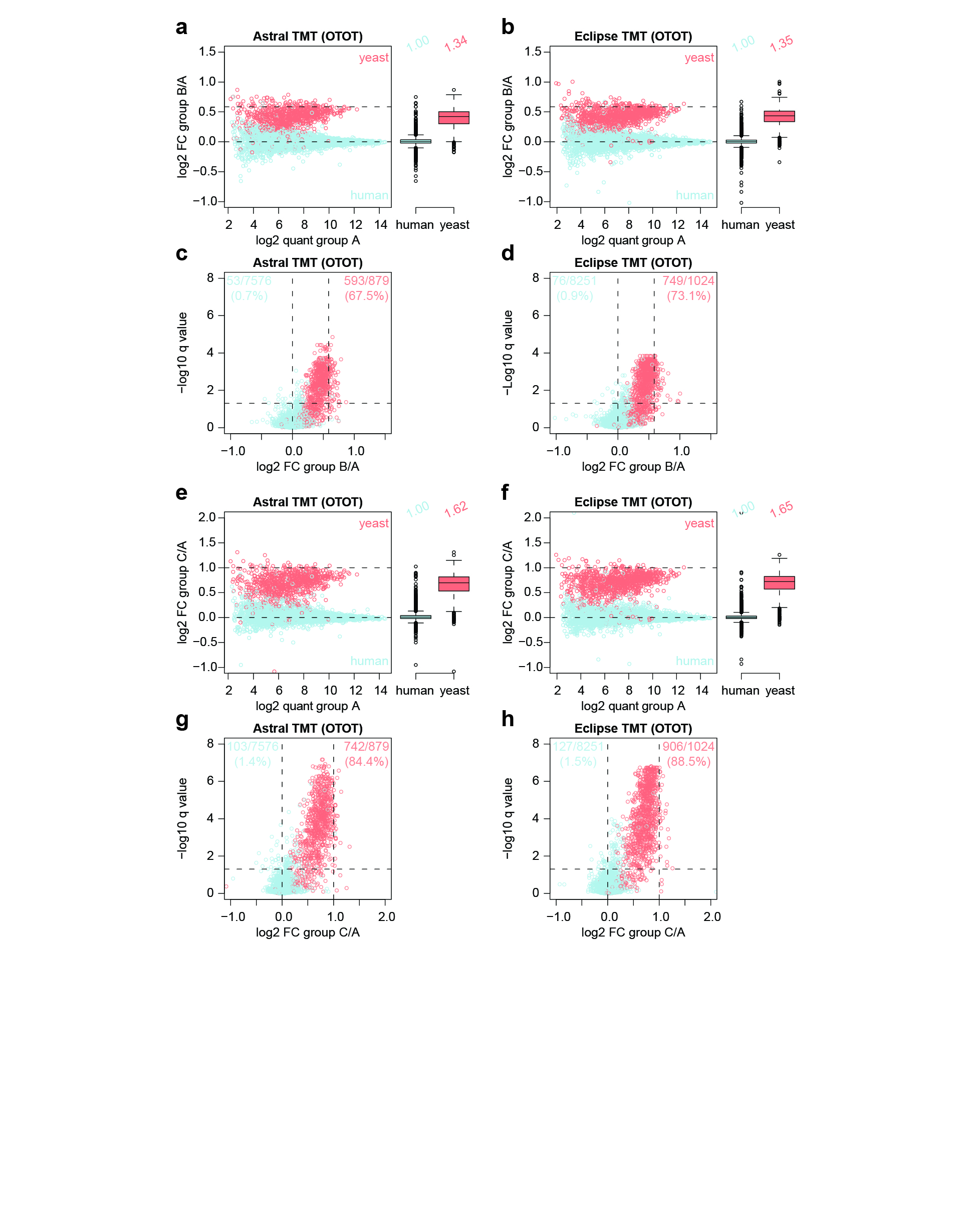


Supplemental Figure 12. Additional assessment of the quantitative accuracy for TMT using Orbitrap for MS2. Scatter plots with boxplots showing measured fold changes for (a) TMT by the Orbitrap Astral with the Orbitrap as the analyzer for MS2 scans, and (b) TMT by the Orbitrap Eclipse with the Orbitrap as the analyzer for MS2 scans, when the theoretical fold change is 1.5. Volcano plots showing the numbers of significant proteins out of the total number of quantified proteins for (c) TMT by the Orbitrap Astral with the Orbitrap as the analyzer for MS2 scans, and (d) TMT by the Orbitrap Eclipse with the Orbitrap as the analyzer for MS2 scans, when the theoretical fold change is 1.5. Scatter plots with boxplots showing measured fold changes for (e) TMT by the Orbitrap Astral with the Orbitrap as the analyzer for MS2 scans, and (f) TMT by the Orbitrap Eclipse with the Orbitrap as the analyzer for MS2 scans, when the theoretical fold change is 2. Volcano plots showing the numbers of significant proteins out of the total number of quantified proteins for (g) TMT by the Orbitrap Astral with the Orbitrap as the analyzer for MS2 scans, and (h) TMT by the Orbitrap Eclipse with the Orbitrap as the analyzer for MS2 scans, when the theoretical fold change is 2.


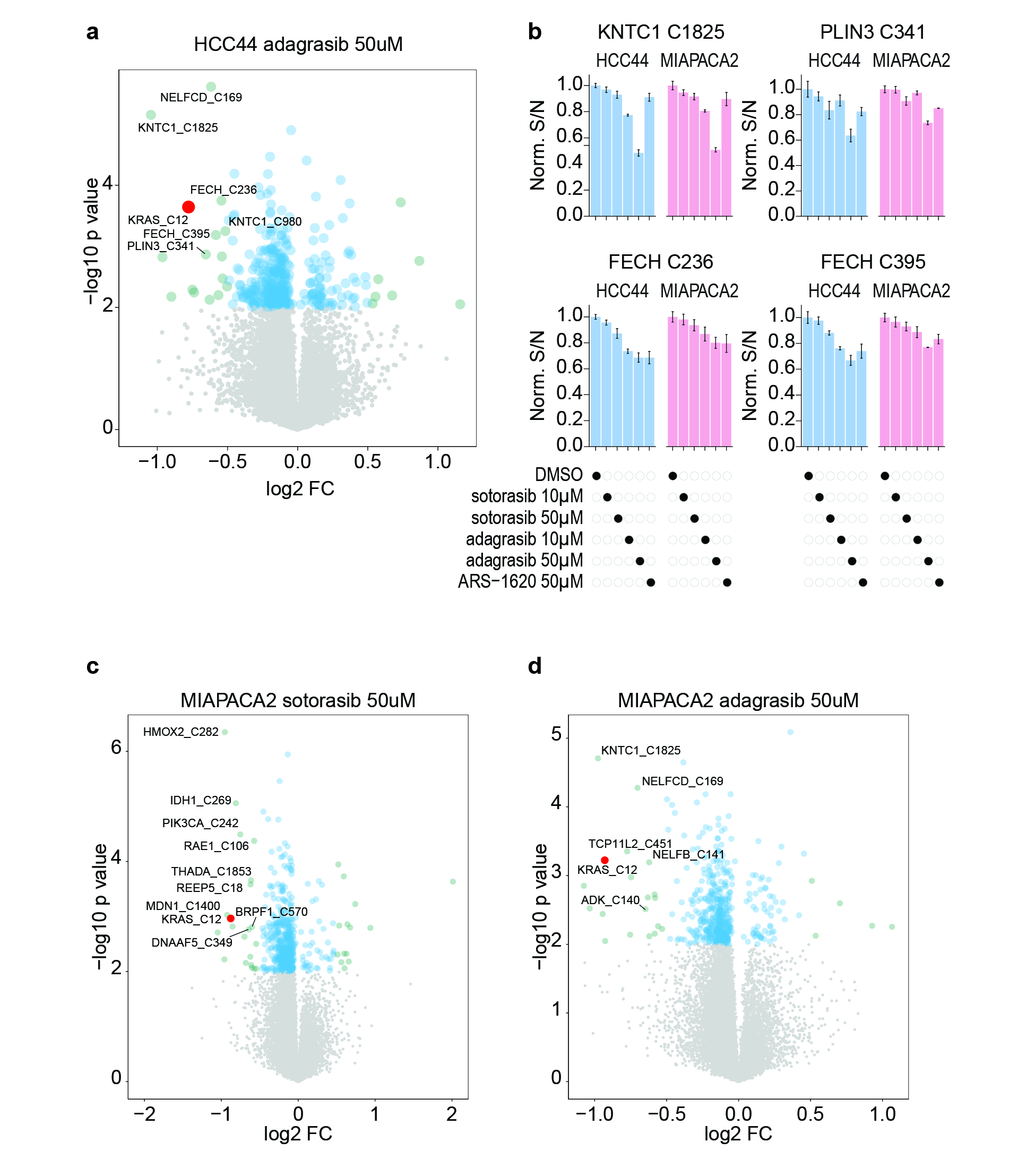


Supplemental Figure 13. Additional assessment of off-targets of KRAS G12C inhibitors by TMT-ABPP on the Astral. (a) Volcano plot showing engagement of cysteine sites with adagrasib in HCC44. (b) Bar plots showing examples of significantly engaged cysteine sites in (a) with 50 µM adagrasib in HCC44. (c) Volcano plot showing engagement of cysteine sites with 50 µM sotorasib in MIAPACA2. (D) Volcano plot showing engagement of cysteine sites with 50 µM adagrasib in MIAPACA2.


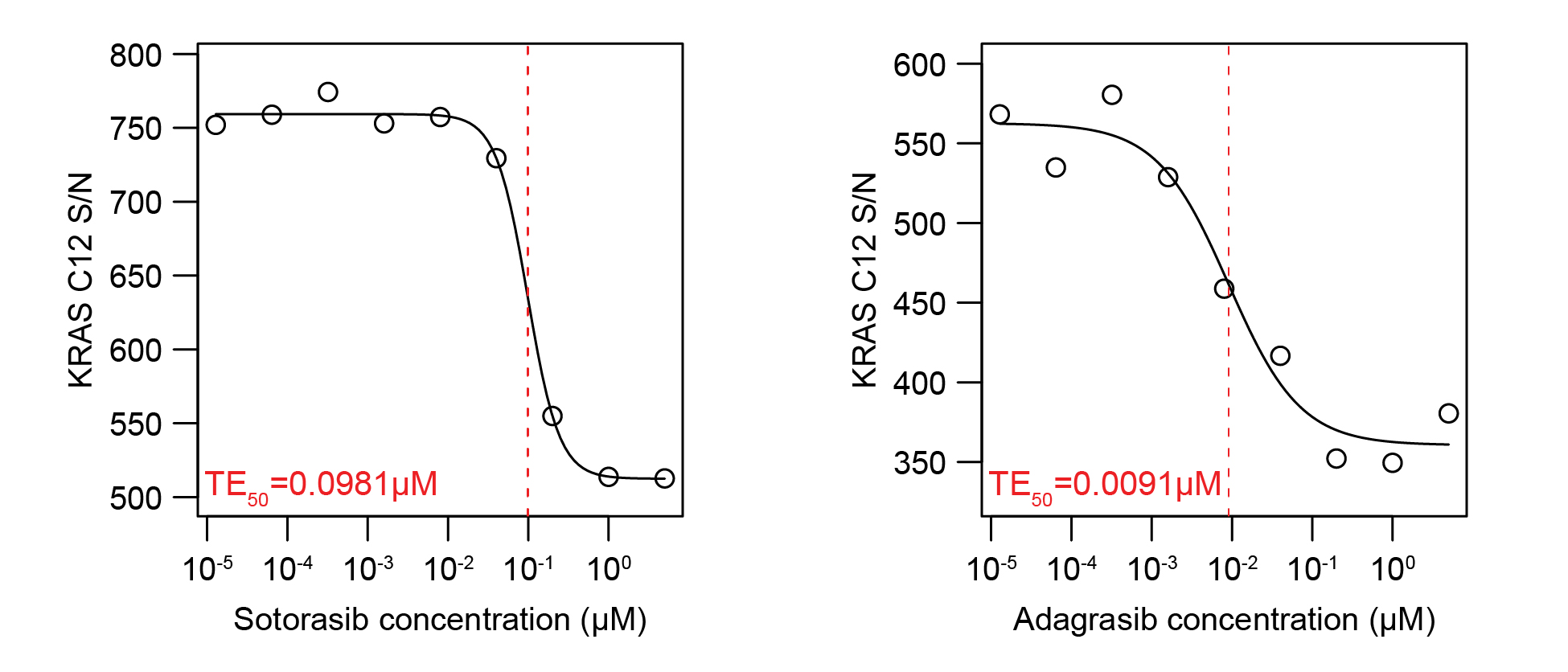


Supplemental Figure 14. Dose response of KRAS C12 to sotorasib and adagrasib. HCC44 native cell lysate was treated by a concentration range of sotorasib and adagrasib from 5 µM to 12.8 pM.
